# Comprehensive Health Assessment Using Risk Prediction for Multiple Diseases Based on Health Checkup Data

**DOI:** 10.1016/j.focus.2024.100277

**Published:** 2024-10-03

**Authors:** Kosuke Yasuda, Shiori Tomoda, Mayumi Suzuki, Toshikazu Wada, Toshiyuki Fujikawa, Toru Kikutsuji, Shintaro Kato

**Affiliations:** 1 NEC Solution Innovators, Ltd., Tokyo, Japan; 2 Kurashiki Central Hospital Preventive Healthcare Plaza, Okayama, Japan; 3 Kurashiki Central Hospital, Okayama, Japan

## Abstract

**Introduction:**

Tools developed to assess individuals’ comprehensive health status would be beneficial for personalized prevention and treatment. This study aimed to develop a set of risk prediction models to estimate the risk for multiple diseases such as heart, blood vessel, brain, metabolic, liver, and kidney diseases using health checkup data only.

**Methods:**

This is a retrospective study that used health checkup data combined with diagnostic information from electronic health records of Kurashiki Central Hospital in Okayama, Japan. All exposure factors were measured at the first health checkup visit, including demographic characteristics, laboratory test results, lifestyle questionnaires, medication use, and medical history. Primary outcomes were the diagnoses of 15 diseases during the follow-up period. Cox proportional hazard regression was applied to develop risk prediction models for heart, blood vessel, brain, metabolic, liver, and kidney diseases. Area under the curve with 4-year risk assessments were performed to evaluate the models.

**Results:**

From January 2012 to September 2022, a total of 92,174 individuals aged 15–96 years underwent general health checkups. The area under the curve of the models in validation datasets was as follows: atrial fibrillation, 0.81; acute myocardial infarction, 0.81; heart failure, 0.76; cardiomyopathy, 0.72; angina pectoris, 0.70; atherosclerosis, 0.82; hypertension, 0.80; cerebral infarction, 0.77; intracerebral hemorrhage, 0.68; subarachnoid hemorrhage, 0.50; type-2 diabetes mellitus, 0.82; hyperlipidemia, 0.70; alcoholic liver disease, 0.91; liver fibrosis, 0.92; and chronic kidney disease, 0.80.

**Conclusions:**

A set of prediction models to estimate multi-disease risk simultaneously from health checkup results may help to assess comprehensive individual health status and facilitate personalized prevention and early diagnosis.

## Highlights

• Comprehensive health assessment would be necessary for effective prevention.
• A set of risk prediction models can predict multiple outcomes simultaneously.
• Multiple prediction models were developed with health checkup and diagnostic data.
• Main outcomes were heart, blood vessel, brain, metabolic, liver, and kidney diseases.
• Modifiable lifestyle risk factors were included in the risk prediction models.

## Introduction

Life expectancy has increased steadily over decades, and the increase in the aging population has raised the incidence of diseases.^1^ Older individuals in Japan are affected by health problems in the last 9.4 years of life.^2^ The continued increase in life expectancy imposes an economic burden on families and the government. Thus, sustainable health care requires a shift from the treatment of established diseases to disease prevention and early diagnosis.^3^ Early lifestyle modification and interventions, including walking, physical activity, and eating habits (e.g., alcohol intake), can prevent diabetes and cardiovascular diseases (CVDs),^4^^,^^5^ thereby reducing health care costs.^6^ Therefore, there is an urgent need to develop a practical risk prediction model using cost-effective tests that are easily accessible, which can help stratify people at risk for developing chronic diseases.

A comprehensive and holistic health evaluation for multiple disease risks would be necessary to understand an individual's overall health status and identify any potential risk factors. As individuals age, they tend to develop multiple chronic diseases.^7^ After age 65 years, 62.8% of the population is affected by 2 or more chronic diseases.^8^ Thus, identifying the disease with the highest risk is needed for effective prevention. However, current risk prediction models do not assess the overall health status and prioritize health problems because they often focus on a single outcome: hypertension,^9^^,^^10^ CVDs,^11^, ^12^, ^13^ diabetes,^14^, ^15^, ^16^ stroke,^17^, ^18^, ^19^ or chronic kidney disease.^20^^,^^21^ Most medical prevention and treatment programs are focused on specific diseases,^22^ which could ignore a hidden disease until it is no longer silent. Although a single disease prediction focuses on a single outcome, a set of risk prediction models can simultaneously predict multiple outcomes by combing such models. This study aimed to develop a set of risk prediction models to estimate the incidence of diseases of multiple organ systems (heart, blood vessels, brain, metabolism, liver, and kidneys) for a general population using only health checkup data.

## Methods

### Study Population

In this retrospective study, 92,174 individuals who visited the Kurashiki Central Hospital Preventive Healthcare Plaza for health checkups were included and were diagnosed at the Kurashiki Central Hospital in Kurashiki City, Japan, between January 2012 and September 2022. Kurashiki Central Hospital is a general hospital with 37 departments covering a medical area of approximately 800,000 people in the western part of Okayama Prefecture. Medical record and health checkup data were merged using common identification number after being deidentified. Most participants had multiple time points of health checkup data. Therefore, the first visit of health checkup data was used as the baseline. The study protocol was approved by the IRB of Kurashiki Central Hospital (IRB Approval Number: 4130). Deidentified data can be made available to researchers whose proposed use of the data is approved.

**Figure 1:**
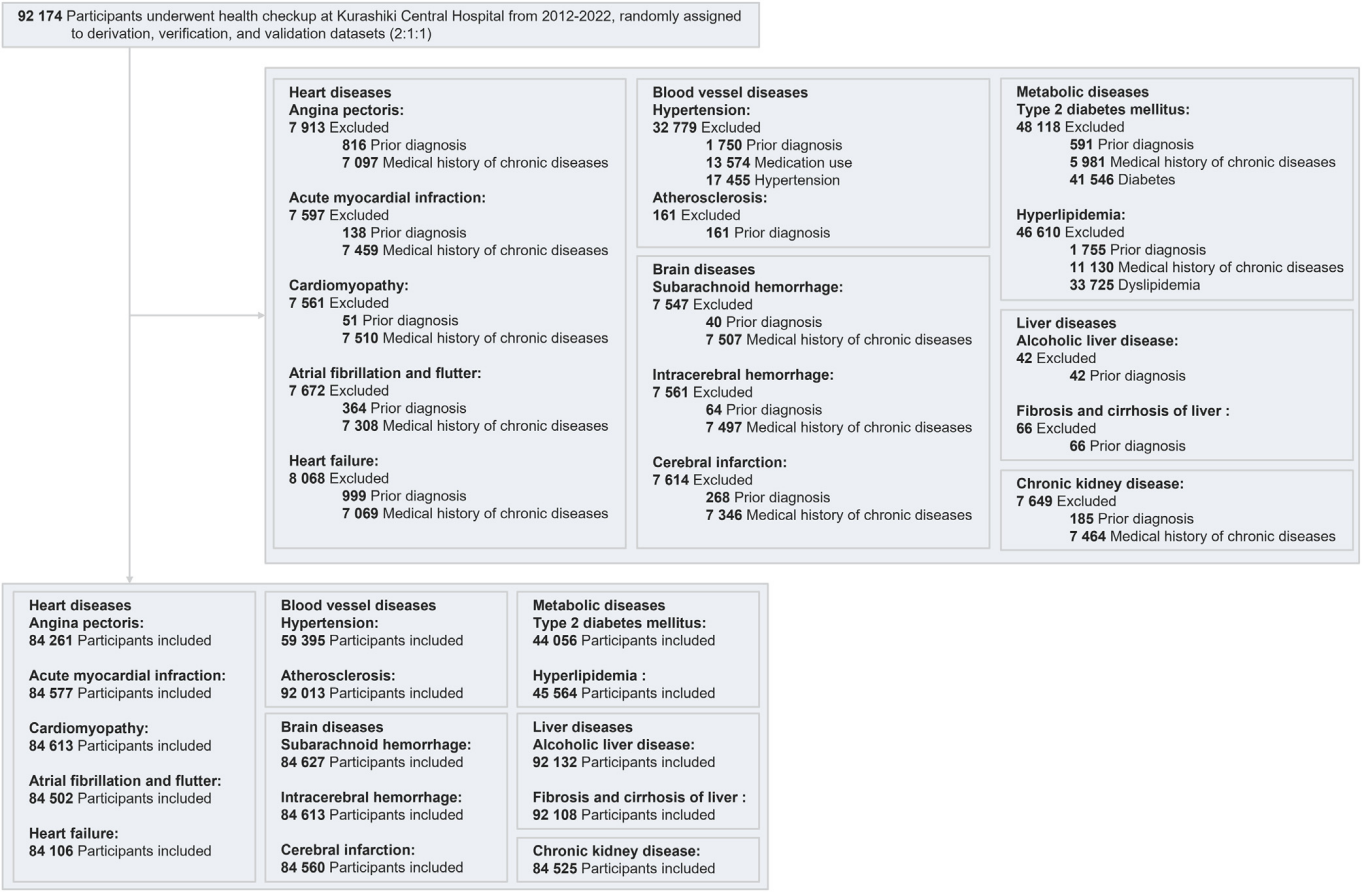
Diagram showing inclusion/exclusion criteria

Different exclusion criteria were set for each disease to consider different levels of disease severity (Appendix Table 1, available online), as the moderate stage of diseases such as hypertension, diabetes, and hyperlipidemia could be related to the incidence of more severe diseases. To develop the risk prediction models for essential (primary) hypertension (HT), type 2 diabetes mellitus (DM), and disorders of lipoprotein metabolism and other lipidemia (HL), participants who were already taking medications (based on self-reports mentioning that they were receiving treatments) already had HT (systolic blood pressure ≥140 mmHg or diastolic blood pressure ≥90 mmHg), DM (fasting plasma glucose ≥126 mg/dL or HbA1c ≥6.5%), or HL (low density lipoprotein cholesterol [LDL] ≥140 mg/dL, high density lipoprotein cholesterol [HDL] <40 mg/dL, or triglyceride ≥150 mg/dL) according to laboratory test results at the baseline health checkups, or lacked this information were excluded. Participants with a history of chronic diseases (stroke, heart disease, and kidney disease), defined as self-reported at baseline health checkups, were excluded from the risk prediction models for severe conditions such as angina pectoris (AP), acute myocardial infarction (MI), cardiomyopathy (CM), atrial fibrillation and flutter (AF), heart failure (HF), subarachnoid hemorrhage (SAH), intracerebral hemorrhage (ICH), cerebral infarction, and chronic kidney disease (CKD). The method of selection of the participants is shown in Figure 1.

### Measures

All exposure factors were measured at the first visit of a health checkup, including demographic characteristics (age, sex, height, weight, BMI, and waist circumference [WC]), laboratory tests (systolic blood pressure, diastolic blood pressure, and γ-glutamyl transpeptidase [γ-GTP], aspartate transaminase [AST], alanine transaminase [ALT], HDL, LDL, triglycerides [TG], fasting plasma glucose [GLU], HbA1c, and uric acid [UA] levels), lifestyle factors (smoking, physical activity, dietary habits, alcohol consumption, and sleeping), medical history (stroke, heart disease, kidney disease, and anemia), and medication use (for HT, DM, and HL). HbA1c was only used to predict DM because of the high rate of missing data. Lifestyle factors, medication use, and medication history were assessed using questionnaires (Appendix Table 2, available online).

Individual endpoints were defined as the first confirmed diagnoses by medical doctors in the electronic health record (EHR) data during the follow-up period. Suspected disease was excluded, and only confirmed diagnoses were considered as endpoints. Each diagnosis was identified according to the ICD-10 terms from the EHR data: E11, DM; E78, HL; I10, HT; I20, AP; I21, MI; I42, CM; I48, AF; I50, HF; I60, SAH; I61, ICH; I63, cerebral infarction; I70, atherosclerosis (AT); K70, alcoholic liver disease (ALD); K74, fibrosis and cirrhosis of the liver (LC); and N18, CKD. When participants had no events (event-free group), the survival time was defined as the period from the first visit for a health checkup to their last visit for a health checkup or the latest diagnosis of other diseases, whichever was later. Therefore, there are 2 types of groups within an event-free group. Appendix Figure 1 shows the schematic describing a typical timeline for survival analysis.

### Statistical Analysis

The participants were randomly categorized into derivation (50%), verification (25%), and validation (25%), datasets for internal validation. Each model was developed using derivation datasets, and its performance was assessed twice using verification and validation datasets. For describing the baseline characteristics, continuous variables are presented as medians (interquartile ranges), and categorical variables are presented as numbers (percentages). To check for selection bias between 2 types of event-free groups, the Wilcoxon rank sum test was performed to compare the follow-up periods. Before model development, continuous variables (age, height, weight, BMI, WC, systolic blood pressure, diastolic blood pressure, γ-GTP, AST, ALT, HDL, LDL, TG, GLU, HbA1c, and UA) were converted to log space with base 10 before analysis because they were relatively log-normally distributed (Appendix Table 3, available online). The Kolmogorov–Smirnov test was performed to check the lognormality. Next, to examine association between various independent variables and outcomes, single-variable Cox proportional hazards regression was used. A stepwise selection with the Akaike information criterion (AIC) for multivariate Cox proportional hazard regression was performed to select variables from all of those. After the variable selection, the prediction models were built with complete cases for selected variables. No imputation methods for missing values were performed in this study. Participants with missing values for selected variables were excluded from the performance evaluation of the prediction models. Baseline hazards and linear predictors of Cox proportional hazards regression were used to calculate the 4-year risk. Model performance was assessed by discrimination and calibration. The area under the curve (AUC) was calculated using the receiver operating characteristic (ROC) curve at 4 years. Event groups were defined as subjects with an event within 4 years, while the event-free group were defined as subjects without an event during the 4-year follow-up period. Harrell's C-index was used to assess discrimination performance over the entire period. The Hosmer-Lemeshow test was performed by dividing the predicted 4-year risks into deciles (10 groups based on percentile ranks) to assess the calibration performance. Spearman's correlations were used to assess the relationship between each pair of relative risks for diseases. Relative risks were calculated by dividing the individual 4-year risk by the median 4-year risk of participants of the same sex and generation (± 5 years). Statistical significance was set at *p*<0.05, unless otherwise noted. All analyses were performed using R, version 4.2.3 (R Foundation for Statistical Computing, Vienna, Austria).^23^

## Results

From January 2012 to September 2022, a total of 92,174 individuals with a median age of 48.29 years (range 15–96 years) underwent health checkups (Appendix Figure 2, available online). Table 1 presents the baseline characteristics of the study population. The number of events during the 10-year follow-up period is summarized in Appendix Table 4. In this study population, the incidence rates of AP, MI, CM, AF, and HF were 38.1, 7.1, 2.5, 21.2, and 47.9 per 10,000 person-years, respectively (Appendix Table 5, available online). The incidence rates of HT and AT were 60.4 and 10.6 per 10,000 person-years, respectively. The incidence rates of SAH, ICH, and cerebral infarction were 1.7, 4.0, and 15.7 per 10,000 person-years, respectively. The incidence rates of DM, HL, ALD, LC, and CKD were 23.6, 48.1, 3.1, 4.2, and 15.0 per 10,000 person-years, respectively. There were significant differences between the follow-up periods of the 2 types of groups within the event-free group (Appendix Table 6, available online).

**Table 1:** Baseline Characteristics of Study Population in the Derivation, Verification, and Validation Datasets

Characteristic	All participants	Derivation	Verification	Validation
	(*n* = 92,174)	(*n* = 46,087)	(*n* = 23,043)	(*n* = 23,044)
Age, years	48.29 (38.92–59.28)	48.21 (38.85–59.21)	48.50 (39.07–59.38)	48.25 (38.88–59.32)
Men, *n* (%)	39,486 (42.8)	19,733 (42.8)	9,832 (42.7)	9,921 (43.1)
Height, cm	162.80 (156.70–169.70)	162.70 (156.70–169.70)	162.70 (156.60–169.60)	162.80 (156.70–169.70)
Weight, kg	59.10 (51.40–68.30)	59.20 (51.40–68.30)	59.10 (51.20–68.30)	59.10 (51.50–68.10)
BMI, kg/m^2^	22.20 (20.10–24.60)	22.20 (20.10–24.60)	22.20 (20.10–24.60)	22.20 (20.10–24.60)
WC, cm	80.00 (73.50–86.60)	80.00 (73.50–86.60)	80.00 (73.60–86.60)	80.00 (73.50–86.70)
Systolic blood pressure, mmHg	119.00 (107.00–132.00)	119.00 (107.00–132.00)	118.00 (107.00–132.00)	119.00 (107.00–132.00)
Diastolic blood pressure, mmHg	74.00 (66.00–82.00)	74.00 (66.00–82.00)	74.00 (66.00–82.00)	74.00 (66.00–82.00)
γ-GTP, U/l	21.00 (14.00–36.00)	21.00 (14.00–36.00)	21.00 (14.00–37.00)	21.00 (14.00–36.00)
AST, U/l	20.00 (17.00–24.00)	20.00 (17.00–24.00)	20.00 (17.00–24.00)	20.00 (17.00–24.00)
ALT, U/l	17.00 (13.00–25.00)	17.00 (13.00–25.00)	17.00 (13.00–25.00)	17.00 (13.00–25.00)
HDL, mg/dl	58.00 (49.00–69.00)	58.00 (49.00–69.00)	58.00 (49.00–69.00)	58.00 (49.00–69.00)
LDL, mg/dl	117.00 (97.00–138.00)	117.00 (97.00–138.00)	117.00 (97.00–138.00)	116.00 (97.00–138.00)
TG, mg/dl	80.00 (57.00–117.00)	80.00 (57.00–117.00)	80.00 (57.00–117.00)	80.00 (56.00–116.00)
GLU, mg/dl	94.00 (89.00–101.00)	94.00 (89.00–101.00)	94.00 (89.00–101.00)	94.00 (89.00–101.00)
HbA1c, %	5.40 (5.20–5.60)	5.40 (5.20–5.60)	5.40 (5.20–5.60)	5.40 (5.20–5.60)
UA, mg/dl	5.10 (4.20–6.20)	5.10 (4.20–6.20)	5.10 (4.20–6.10)	5.10 (4.20–6.10)
Smoking, *n* (%)	15,178 (17.1)	7,607 (17.2)	3,729 (16.8)	3,842 (17.4)
Exercise, *n* (%)	18,705 (20.7)	9,424 (20.9)	4,642 (20.6)	4,639 (20.6)
Walking speed, *n* (%)	36,287 (40.2)	18,219 (40.4)	9,109 (40.4)	8,959 (39.8)
Weight gain from age 20 years, *n* (%)	28,219 (31.5)	14,152 (31.5)	7,127 (31.8)	6,940 (31.0)
Weight change in past 1 year, *n* (%)	18,995 (26.4)	9,608 (26.7)	4,727 (26.2)	4,660 (26.0)
Drinking frequency, *n* (%)				
Rarely	42,363 (47.9)	21,179 (47.9)	10,550 (47.7)	10,634 (48.1)
Sometimes	26,577 (30.0)	13,278 (30.0)	6,680 (30.2)	6,619 (29.9)
Everyday	19,517 (22.1)	9,785 (22.1)	4,882 (22.1)	4,850 (21.9)
Drinking volume, *n* (%)				
<180 ml	33,179 (57.0)	16,677 (57.1)	8,265 (56.6)	8,237 (57.0)
180–360 ml	16,075 (27.6)	8,009 (27.4)	4,061 (27.8)	4,005 (27.7)
360–540 ml	6,759 (11.6)	3,378 (11.6)	1,697 (11.6)	1,684 (11.7)
>540 ml	2,226 (3.8)	1,118 (3.8)	584 (4.0)	524 (3.6)
Eating late dinner, *n* (%)	23,337 (25.9)	11,748 (26.0)	5,754 (25.5)	5,835 (25.9)
Skipping breakfast, *n* (%)	14,393 (16.0)	7,280 (16.1)	3,577 (15.9)	3,536 (15.7)
Eating snacks, *n* (%)	11,860 (16.5)	5,996 (16.6)	2,972 (16.5)	2,892 (16.2)
Sleeping well, *n* (%)	58,117 (64.5)	28,934 (64.2)	14,488 (64.3)	14,695 (65.3)
Medication use, *n* (%)				
Hypertension	11,640 (13.1)	5,740 (12.9)	3,036 (13.6)	2,864 (12.9)
Diabetes mellitus	3,021 (3.4)	1,524 (3.4)	750 (3.4)	747 (3.4)
Hyperlipidemia	8,682 (9.8)	4,368 (9.8)	2,125 (9.6)	2,189 (9.9)
Medical history, *n* (%)				
Stroke	1,290 (1.5)	653 (1.5)	330 (1.5)	307 (1.4)
Heart disease	2,438 (2.8)	1,217 (2.7)	621 (2.8)	600 (2.7)
Kidney disease	187 (0.2)	93 (0.2)	41 (0.2)	53 (0.2)

Continuous variables are expressed as median (IQR].

Categorical variables are expressed as the number of subjects and proportions (percentages).

ALT, alanine transaminase; AST, aspartate transaminase; γ-GTP, γ-glutamyl transpeptidase; GLU, fasting plasma glucose; hbHDL, high density lipoprotein cholesterol; LDL, low density lipoprotein cholesterol; TG, triglycerides; UA, uric acid; WC, waist circumference.

To examine the association between various independent variables and outcomes, single-variable Cox proportional hazards regression was performed (Appendix Figures 3–17, available online). The volcano plots showed that aging was a common risk factor for heart (AP, MI, CM, AF, and HF), blood vessel (HT and AT), brain (cerebral infarction and ICH), metabolism (DM and HL), liver (ALD and LC), and kidney diseases (CKD). Systolic and diastolic blood pressure were commonly associated with CVDs (AF, MI, HF, CM, AP, cerebral infarction, and ICH), although there was no significant association found between health checkup results and SAH. The liver function tests (γ-GTP, AST, and ALT) were associated with liver disease (ALD and LC) as well as metabolic diseases (DM and HL). Next, multivariate Cox proportional hazards regression with selected variables determined by stepwise variable selection was performed for building the risk prediction models. The multivariate-adjusted regression coefficients for each disease are presented in Appendix Tables 7–12, available online. After the stepwise variable selection, blood pressure was commonly retained in the models for heart (AP, MI, CM, AF, and HF), blood vessel (HT and AT), brain (SAH, ICH, and cerebral infarction), and kidney (CKD) diseases. Concerning the lifestyle risk factors, fast walking speed was selected for a lower incidence in the models of heart (AP, MI, AF, and HF), blood vessel (HT), and brain diseases (SAH and ICH). Drinking alcohol (>540 mL) was incorporated with the models of HT, DM, and ALD. The models for heart (AP), blood vessel (AT), and brain (cerebral infarction) disease risks included smoking status.

**Figure 2:**
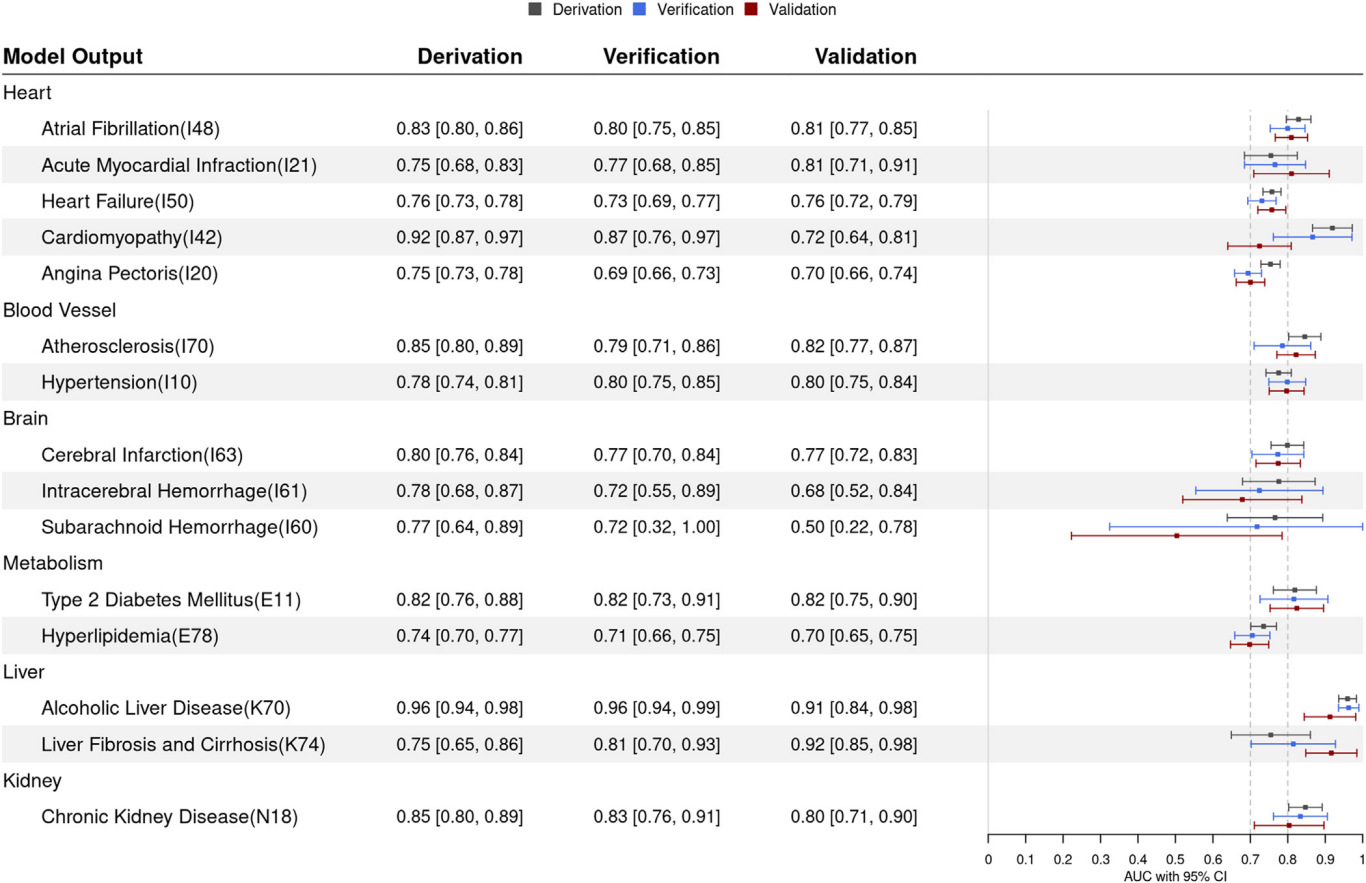
Discrimination performance of risk prediction models AUCs and 95% CIs for 4-year risk of heart, blood vessel, brain, metabolic, liver, and kidney diseases in derivation, verification, and validation datasets. AUC, area under the curve

ROC curve analysis was performed to evaluate the performance of the risk prediction models (Figure 2). The prediction models for liver diseases showed higher AUCs for 4-year risks in the validation datasets (ALD, 0.91 [95% CI=0.84, 0.98] and LC, 0.92 [95% CI=0.85, 0.98]). The 4-year AUCs for 5 heart diseases ranged from 0.81 [95% CI=0.77, 0.85] for AF to 0.70 [95% CI=0.66, 0.74] for AP. The discriminative abilities of the prediction model for blood vessel, metabolic, and kidney diseases were as follows: AT, 0.82 [95% CI=0.77, 0.87]; HT, 0.80 [95% CI=0.75, 0.84]; DM, 0.82 [95% CI=0.75, 0.90]; HL, 0.70 [95% CI=0.65, 0.75]; and CKD, 0.80 [95% CI=0.71, 0.90]. The prediction model for cerebral infarction showed a relatively high AUC (0.77 [95% CI=0.72, 0.83]), whereas the prediction models for ICH and SAH showed lower discriminatory performance (ICH, 0.68 [95% CI=0.52, 0.84] and SAH, 0.5 [95% CI=0.22, 0.78]). The 4-year AUCs for CM ranged from 0.92 [95% CI=0.87, 0.97] to 0.72 [95% CI=0.64, 0.81]. Harrell's C-index was assessed for discrimination performance over the entire period (Appendix Table 13, available online). Harrell's C-index values were ≥0.7 in all prediction models except for HL, ICH, and SAH with validation data. Moreover, the calibration of the risk prediction models, a measure of agreement between observed and predicted events in 4 years, was assessed with the Hosmer–Lemeshow test (Appendix Table 14, available online). The risk prediction models for MI, CM, AT, cerebral infarction, ICH, SAH, DM, HL, LC, and CKD were well calibrated, whereas the prediction models for AF, HF, AP, HT, and ALD were not (Hosmer–Lemeshow test, AF, *p*=0.037; HF, *p*=0.001; AP, *p*<0.001, HT; *p*=0.003; and ALD, *p*=0.002). Although this study defined the event-free group as the last visit of a health checkup or diagnosis of other diseases, the 2 event-free groups were similar in the distribution of the 4-year risks for all diseases, and their risks were significantly lower than those of the event group (Appendix Table 18, available online).

For the MI risk prediction model, the retained variables were age, systolic blood pressure, diabetes, weight change, walking speed, sex, UA, γ-GTP, ASL, LDL, hyperlipidemia, ALT, and HDL as predictors (Appendix Table 7, available online). The AUCs for MI were 0.75 [95% CI=0.68, 0.83], 0.77 [95% CI=0.68, 0.85], and 0.81 [95% CI=0.71, 0.91] in derivation, verification, and validation data, respectively (Figure 2). The Hosmer-Lemeshow tests for the MI risk prediction model indicated an acceptable goodness of fit (*P*=0.224, 0.075, and 0.615 in derivation, verification, and validation data, respectively) (Appendix Table 14, available online).

**Figure 3:**
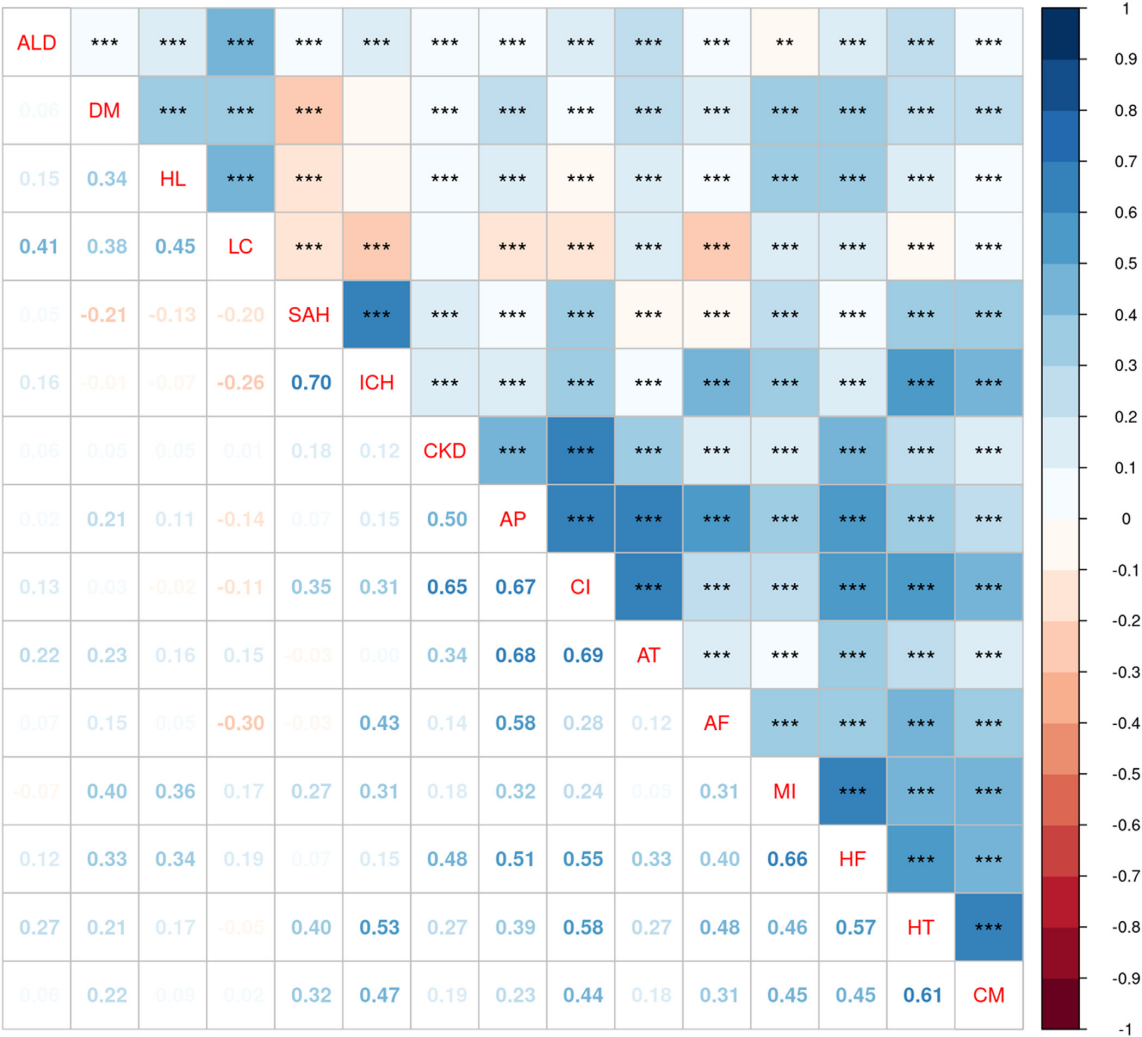
Relationship among risk scores for diseases of multiple organ systems Correlation matrix heatmap of relative risk scores (*n*=92,174 participants). For each disease pair, participants with missing covariate values to predict risk scores were excluded. Numbers are Spearman's correlation coefficients for each pair of risk scores for the 15 diseases. Bonferroni-corrected *P*-values are reported. ***P<0.001, **P<0.01. AF, atrial fibrillation and flutter; ALD, alcoholic liver disease; AP, angina pectoris; AT, atherosclerosis; CI, cerebral infarction; CKD, chronic kidney disease; CM, cardiomyopathy; DM, type-2 diabetes mellitus; HF, heart failure; HL, disorders of lipoprotein metabolism and other lipidemia; HT, essential (primary) hypertension; ICH, intracerebral hemorrhage; LC, fibrosis and cirrhosis of the liver; MI, acute myocardial infarction; SAH, subarachnoid hemorrhage

To test whether disease risks were associated with each other in individuals, Spearman's correlation analysis was performed (Figure 3). Individuals with a higher risk of developing HT were more likely to have heart (CM, Spearman 0.61, *p*<0.001 and HF, Spearman 0.57, *p*<0.001) and brain (cerebral infarction, Spearman 0.58, *p*<0.001 and ICH, Spearman 0.53, *p*<0.001) diseases. AT was highly correlated with cerebral infarction and AP (cerebral infarction, Spearman 0.69, *p*<0.001, and AP, Spearman 0.68, *p*<0.001). ICH was highly correlated with SAH (Spearman 0.70, *p*<0.001). MI was associated with HF (Spearman 0.66, *p*<0.001), and AP was associated with HF (Spearman 0.51, *p*<0.001). Interactions between different organs were also found for heart and brain diseases (cerebral infarction vs HF, Spearman 0.55, *p*<0.001), heart and kidney diseases (AP vs CKD, Spearman 0.50, *p*<0.001), and kidney and brain diseases (CKD vs cerebral infarction, Spearman 0.65, *p*<0.001); however, liver diseases (ALD and LC) had relatively low correlations with diseases of other organs.

## Discussion

This study developed a set of risk prediction models for multiple diseases, including for 5 heart, 2 blood vessel, 3 brain, 2 metabolic, 2 liver, and 1 kidney diseases. The study identified clinical and lifestyle risk factors, data which are readily available and practical to use, in data obtained from the general Japanese population. The prediction models may help individuals become aware of their own health risks and incorporate a healthy lifestyle for disease prevention.

The study performed internal validation and found that the discriminative abilities of our models were comparable to or higher than those of previous clinical models.^9^^,^^11^^,^^12^^,^^14^^,^^20^ According to published literature, an AUC between 0.7 and 0.8 is considered to indicate acceptable discrimination.^24^^,^^25^ Noble et al.^26^ reviewed 94 diabetes risk models with AUCs ranging from 0.62 to 0.91. The discriminative ability of the presented DM model revealed high accuracy with an AUC of 0.82 in the validation dataset. Among the 15 models developed through the validation datasets, 11 models showed acceptable discrimination, with an AUC greater than 0.7 for heart (AF, MI, and HF), blood vessel (AT and HT), brain (cerebral infarction), metabolic (DM and HL), liver (ALD and LC), and kidney (CKD) diseases in both verification and validation datasets. The CM and SAH models seemed to be overfitting, as there were differences in the AUCs among derivation, verification, and validation datasets. The predictive power was enhanced by adding HbA1c to the DM model^16^ and estimated glomerular filtration rate (eGFR) to the CKD model.^20^ The authors did not include data pertaining to biomarkers such as eGFR and N-terminal pro B-type natriuretic peptide, which are not usually assessed during general health checkups. The addition of biomarkers to our prediction model can further improve the accuracy.

Selected variables in each prediction model were similar to previous studies.^27^, ^28^, ^29^ For example, compared with the Suita score, which is a coronary heart disease risk,^27^ the MI risk prediction model of this study shared common risk factors, such as age, systolic blood pressure, diabetes, and LDL. However, the MI risk prediction model did not include current smoking because of unimprovement of AIC values. In addition, the *p*-value of smoking was the highest among the significant variables with single variable analysis, even though current smoking was associated with MI incidence (Appendix Figure 4, available online). One of possible reasons could be that the questionnaire in this study did not consider former smokers associated with a higher risk of MI,^29^ as it only asked for current smoking status (Appendix Table 2, available online). As for other lifestyle risk factors, the MI risk prediction model included walking speed instead of regular exercise reported in a previous study,^30^ because both variables are related to physical activity. This study also revealed that moderate alcohol consumption was associated with a lower risk of DM, the same as previous studies.^31^

The newly developed risk prediction models included some modifiable lifestyle risk factors, which could suggest that lifestyle modification can lower disease risk in an individual. Subjective walking speed, a marker of intensity of physical activity, is associated with the occurrence of MI, HF, AP, and stroke.^32^ Similarly, the study found that subjective walking speed was associated with the risk of developing heart diseases (AF, MI, and HF) as well as metabolic disease (DM). These findings, and those of previous studies,^13^^,^^19^^,^^32^^,^^33^ highlight the potential clinical utility of subjective walking speed as a simple predictive indicator for CVD prevention. Furthermore, current smoking status was associated with a higher risk of blood vessel (AT), heart (AP), and brain (cerebral infarction) diseases. Excessive alcohol consumption also increases the risks of HT, DM, and ALD. WC, a simple method for assessing abdominal adiposity, is straightforward to use in clinical situations. Here, the study found that a large WC could indicate an increased risk of HT, DM, and LC. After the stepwise variable selection, the risk prediction models retained variables with weak associations between lifestyle factors and outcomes because these variables contributed to improved AIC values despite the insignificance. For example, insignificant variables such as skipping breakfast and having a late dinner were associated with an increased DM risk (Appendix Table 10, available online). Thus, these risk prediction models can support the practice of developing a healthy lifestyle to reduce the risk of chronic conditions.

Regular health checkups have the potential to provide comprehensive health assessment for the simultaneous evaluation of multiple disease risks. In the Japanese workplace, employers are required to schedule annual health checkups for workers in accordance with industrial safety and health laws. This study developed a set of risk prediction models for multiple diseases based on data obtained from routine health checkups. The main outcomes included both moderate and severe disease. Moderate stages of diseases such as HT, DM, and HL are the major risk factors for developing atherosclerotic plaques, which are the main underlying pathology for many CVDs.^34^ The correlation analysis of multiple risk scores showed similarities between diseases based on underlying biological processes. These results are consistent with the notion that AT is the main underlying pathology for many CVDs.^34^ HF is a potential and common complication of MI, consistent with the results of previous studies.^35^ In addition, the study found an association among risk scores for diseases of different organs, including heart and kidney diseases (AP vs CKD, Spearman 0.50, *p*<0.001) and kidney and brain diseases (CKD vs cerebral infarction, Spearman 0.65, *p*<0.001). The prediction models for AP, CI, and CKD shared the risk factors, such as systolic blood pressure, UA, GLU, and diabetes medication. In contrast, the occurrence of liver diseases was relatively independent, suggesting that focusing on a single disease could fail to notice other disease risks. The comprehensive assessment of multiple risks across the different diseases could aid physicians in recommending appropriate specialists and advanced examinations. For instance, physicians could recommend a visit to a cardiologist for individuals at a higher risk of heart disease, potentially leading to earlier detection.

The major strengths of this study include its longitudinal design, relatively large sample size, and rigorous feature selection methods. Health check-up data combined with diagnostic information from EHRs provide a unique opportunity to directly examine the effects of clinical and lifestyle risk factors on health outcomes. Because of the similarity of averages of health checkup results between this study and the Japanese population (mean BMI: 22.7 vs 23.1, mean WC: 80.7 vs 82.6, mean GLU: 98 vs 98, mean HbA1c: 5.5 vs 5.3, mean systolic blood pressure: 121 vs 126, mean diastolic blood pressure: 75 vs 77, mean TG: 98 vs 121, mean HDL: 60 vs 63, and mean LDL: 119 vs 127 in our study and Japanese population, respectively),^36^ these risk models, which used easily accessible and practical variables, could have a potential to be applicable to the general Japanese population. However, the study has limitations. First, our results were not externally validated in independent cohorts. This study had a relatively large sample size, which enabled the use of verification and validation datasets for checking the extent of the misfit of the prediction model. Second, the discrimination of the prediction models for SAH and ICH was relatively low, possibly because of the small number of events. Third, participants diagnosed at other hospitals could not be included in this study, which may have underestimated the incidence rates. Fourth, this study was conducted at a single institution, which might cause selection bias because of the proximity and accessibility of health care services. However, most of the participants were residents in the catchment area of the Kurashiki Central Hospital and could access its services within a 1-hour car ride. Furthermore, given that there were only 2 core general hospitals in this catchment area, there could not be any significant barriers or differences in accessing the hospital. Fifth, in this retrospective study, most of the baseline health checkup data were collected between 2012 and 2013 (2012: 38%, 2013: 10%). Thus, the population could relatively reflect the age structure of the Japanese population in 2012–2013, but not the current one. Appendix Figure 1 shows that the male population had 2 peaks around ages 40 and 64, which were relatively close to the age structure of Japan in 2012. On the other hand, there was no observation of 2 peaks with the female population (Japan's Ministry of Health, Labor, and Welfare, www.stat.go.jp/english/data/jinsui/2012np/index.html), suggesting the presence of a selection bias in our study. Therefore, a validation study needs to be conducted for better understanding about the reproducibility and generalizability of our prediction models to other populations.

## Conclusions

In conclusion, the risk prediction models integrated important clinical and lifestyle risk factors to predict the individual risk of 15 diseases of multiple organ systems. Patients at high risk should be flagged for screening and aggressive treatment of risk factors. A set of risk prediction models for the 6 organ systems has the potential to help individuals assess their overall health and change their lifestyles in a timely manner for disease prevention.

## Appendix Supplementary materials

Image, application 1

## Footnotes

Supplementary material associated with this article can be found in the online version at doi:10.1016/j.focus.2024.100277.
