# Supplementary material for: Comprehensive Health Assessment Using Risk Prediction for Multiple Diseases Based on Health Checkup Data
Source: AJPM Focus. 2024 Oct 3;3(6):100277. doi: 10.1016/j.focus.2024.100277 (PMC11567062; doi:10.1016/j.focus.2024.100277)
Supplement: Supplementary file 1 [file mmc1.docx]

**Appendix Material**

**Table of contents**

| **No.** | **Content** | **Page no.** |
| --- | --- | --- |
| 1 | Appendix Table 1 | 2 |
| 2 | Appendix Table 2 | 4 |
| 3 | Appendix Table 3 | 5 |
| 4 | Appendix Table 4 | 7 |
| 5 | Appendix Table 5 | 9 |
| 6 | Appendix Table 6 | 10 |
| 7 | Appendix Table 7 | 11 |
| 8 | Appendix Table 8 | 12 |
| 9 | Appendix Table 9 | 13 |
| 10 | Appendix Table 10 | 14 |
| 11 | Appendix Table 11 | 15 |
| 12 | Appendix Table 12 | 16 |
| 13 | Appendix Table 13 | 17 |
| 14 | Appendix Table 14 | 18 |
| 15 | Appendix Figure 1 | 19 |
| 16 | Appendix Figure 2 | 20 |
| 17 | Appendix Figure 3 | 21 |
| 18 | Appendix Figure 4 | 22 |
| 19 | Appendix Figure 5 | 23 |
| 20 | Appendix Figure 6 | 24 |
| 21 | Appendix Figure 7 | 25 |
| 22 | Appendix Figure 8 | 26 |
| 23 | Appendix Figure 9 | 27 |
| 24 | Appendix Figure 10 | 28 |
| 25 | Appendix Figure 11 | 29 |
| 26 | Appendix Figure 12 | 30 |
| 27 | Appendix Figure 13 | 31 |
| 28 | Appendix Figure 14 | 32 |
| 29 | Appendix Figure 15 | 33 |
| 30 | Appendix Figure 16 | 34 |
| 31 | Appendix Figure 17 | 35 |
| 32 | Appendix Figure 18 | 36 |

**Appendix Table 1. Exclusion criteria**

| **ICD-10 code** | **Name** | **Abbreviation** | **Exclusion criteria** |
| --- | --- | --- | --- |
| E11 | Type 2 diabetes mellitus | DM | Prior diagnosis of DM  Treatment of DM: Yes  126 mg/dL ≥ GLU  6.5% ≥ HbA1c |
| E78 | Disorders of lipoprotein metabolism and other lipidemias | HL | Prior diagnosis of HL  Treatment of HL: Yes  LDL ≥ 140 mg/dL  HDL < 40 mg/dL  TG ≥ 150 mg/dL |
| I10 | Essential (primary) hypertension | HT | Prior diagnosis of HT  Treatment of HT: Yes  Systolic blood pressure ≥ 140 mmHg Diastolic blood pressure ≥ 90 mmHg |
| I20 | Angina pectoris | AP | Prior diagnosis of AP  Medical history of heart disease: Yes  Medical history of stroke: Yes  Medical history of kidney disease: Yes |
| I21 | Acute myocardial infarction | MI | Prior diagnosis of MI  Medical history of heart disease: Yes  Medical history of stroke: Yes  Medical history of kidney disease: Yes |
| I42 | Cardiomyopathy | CM | Prior diagnosis of CM  Medical history of heart disease: Yes  Medical history of stroke: Yes  Medical history of kidney disease: Yes |
| I48 | Atrial fibrillation and flutter | AF | Prior diagnosis of AF  Medical history of heart disease: Yes  Medical history of stroke: Yes  Medical history of kidney disease: Yes |
| I50 | Heart failure | HF | Prior diagnosis of HF  Medical history of heart disease: Yes  Medical history of stroke: Yes  Medical history of kidney disease: Yes |
| I60 | Subarachnoid hemorrhage | SAH | Prior diagnosis of SAH  Medical history of heart disease: Yes  Medical history of stroke: Yes  Medical history of kidney disease: Yes |
| I61 | Intracerebral hemorrhage | ICH | Prior diagnosis of ICH  Medical history of heart disease: Yes  Medical history of stroke: Yes  Medical history of kidney disease: Yes |
| I63 | Cerebral infarction | CI | Prior diagnosis of CI  Medical history of heart disease: Yes  Medical history of stroke: Yes  Medical history of kidney disease: Yes |
| I70 | Atherosclerosis | AT | Prior diagnosis of AT |
| K70 | Alcoholic liver disease | ALD | Prior diagnosis of ALD |
| K74 | Fibrosis and cirrhosis of liver | LC | Prior diagnosis of LC |
| N18 | Chronic kidney disease | CKD | Prior diagnosis of CKD  Medical history of heart disease: Yes  Medical history of stroke: Yes  Medical history of kidney disease: Yes |

ICD-10, the International Statistical Classification of Diseases, Version 10; GLU, fasting plasma glucose; HbA1c, hemoglobin A1c; LDL, low density lipoprotein; HDL, high density lipoprotein; TG, triglyceride

**Appendix Table 2. Health checkup questionnaire**

| **Category** | **No.** | **Questions** | **Responses** |
| --- | --- | --- | --- |
| Smoking status | 1 | Are you currently a heavy smoker? A heavy smoker refers to those who have smoked a total of over 100 cigarettes over a period of 6 months and have been smoking over the past month. | Yes or no |
| Physical activity | 2 | Are you in a habit of doing exercise to sweat lightly for over 30 minutes a time, 2 times weekly, for over a year? | Yes or no |
|  | 3 | Is your walking speed faster than the speed of those of your age and sex? | Yes or no |
| Body weight change | 4 | Have you gained or lost more than 3 kg in the past year? | Yes or no |
|  | 5 | Have you gained over 10 kg from your weight at age 20 years? | Yes or no |
| Drinking frequency | 6 | How often do you drink (sake, shochu, beer, wine, whisky, or brandy, etc.)? | Every day, sometimes, or rarely drink (cannot drink) |
| Drinking volume | 7 | How much do you drink per day (1 cup of sake [180 mL], 1 medium beer bottle [500 mL], 25% shochu [110 mL], a double shot of whiskey [60 mL], 2 glasses of wine [240 mL])? | Less than 180 mL, 180 – 360 mL, 360 – 540 mL, or more than 540 mL |
| Dietary habits | 8 | Do you eat supper 2 hours before bedtime more than 3 times a week? | Yes or no |
|  | 9 | Do you skip breakfast more than 3 times a week? | Yes or no |
|  | 10 | Do you eat snacks after supper more than 3 times a week? | Yes or no |
| Subjective sleep quality | 11 | Do you sleep well and enough? | Yes or no |
| Medication use |  | Are you taking the following medicines at present? |  |
|  | 12 | Medication to reduce blood pressure | Yes or no |
|  | 13 | Medication to reduce blood sugar or insulin injection | Yes or no |
|  | 14 | Medication to reduce your level of cholesterol or of neutral fat. | Yes or no |
| Medical history | 15 | Have you ever been told by the doctor you have had a stroke (cerebral hemorrhage, brain infarction, etc.) and received treatment? | Yes or no |
|  | 16 | Have you ever been told by the doctor you have a heart disease (angina pectoris, myocardial infarction, etc.) and received treatment? | Yes or no |
|  | 17 | Have you ever been diagnosed as having chronic kidney disease or kidney failure and received treatment (dialysis therapy)? | Yes or no |

**Appendix Table 3. Normality test**

|  |  | **Original value** | | **Log-transformed value** | |
| --- | --- | --- | --- | --- | --- |
| **Variables** | **Relatively distributed in log-normal distribution comparing to KS distance** | **KS distance** | ***p* value** | **KS distance** | ***p* value** |
| Age | FALSE | 0.0436 | <0.001 | 0.0563 | <0.001 |
| Height | TRUE | 0.0367 | <0.001 | 0.0294 | <0.001 |
| Weight | TRUE | 0.0549 | <0.001 | 0.0261 | <0.001 |
| HbA1c | TRUE | 0.1998 | <0.001 | 0.1670 | <0.001 |
| HDL | TRUE | 0.0533 | <0.001 | 0.0244 | <0.001 |
| LDL | TRUE | 0.0356 | <0.001 | 0.0331 | <0.001 |
| TG | TRUE | 0.1765 | <0.001 | 0.0388 | <0.001 |
| γ-GTP | TRUE | 0.2783 | <0.001 | 0.0897 | <0.001 |
| AST | TRUE | 0.2232 | <0.001 | 0.0962 | <0.001 |
| ALT | TRUE | 0.1933 | <0.001 | 0.0723 | <0.001 |
| WC | TRUE | 0.0394 | <0.001 | 0.0165 | <0.001 |
| BMI | TRUE | 0.0648 | <0.001 | 0.0322 | <0.001 |
| Systolic blood pressure | TRUE | 0.0513 | <0.001 | 0.0271 | <0.001 |
| Diastolic blood pressure | TRUE | 0.0449 | <0.001 | 0.0235 | <0.001 |
| GLU | TRUE | 0.1832 | <0.001 | 0.1334 | <0.001 |
| UA | TRUE | 0.0485 | <0.001 | 0.0407 | <0.001 |

The Kolmogorov-Smirnov (KS) test was performed to check the normality using the derivation data (n= 46 087). Abbreviations: ALT, alanine transaminase; AST, aspartate transaminase; BMI, body mass index; γ-GTP, γ-glutamyl transpeptidase; GLU, fasting plasma glucose; HbA1c, Hemoglobin A1c; HDL, high density lipoprotein cholesterol; LDL, low density lipoprotein cholesterol; TG, triglycerides; UA, uric acid; WC, waist circumference.

**Appendix Table 4. Number of events during the follow-up period**

|  |  | Derivation |  |  | Verification |  |  | Validation |  |
| --- | --- | --- | --- | --- | --- | --- | --- | --- | --- |
|  | Participants, No. | Events, No. (%) | Follow-up duration, mean [SD], days | Participants, No. | Events, No. (%) | Follow-up duration, mean [SD], days | Participants, No. | Events, No. (%) | Follow-up duration, mean [SD], days |
| **Heart** |  |  |  |  |  |  |  |  |  |
| Atrial fibrillation (I48) | 42237 | 390 (0.9) | 1696.1 (1394.3) | 21137 | 238 (1.1) | 1711.6 (1396.2) | 21128 | 207 (1.0) | 1696.4 (1393) |
| Acute myocardial infraction (I21) | 42274 | 146 (0.3) | 1703.8 (1397.2) | 21160 | 65 (0.3) | 1721.1 (1400.2) | 21143 | 69 (0.3) | 1705.2 (1395.2) |
| Heart failure (I50) | 42038 | 934 (2.2) | 1679.5 (1391.1) | 21044 | 468 (2.2) | 1694.1 (1393.9) | 21024 | 456 (2.2) | 1682.8 (1389.5) |
| Cardiomyopathy (I42) | 42293 | 49 (0.1) | 1705.5 (1397.5) | 21169 | 22 (0.1) | 1723.4 (1400.5) | 21151 | 28 (0.1) | 1706.3 (1395.6) |
| Angina pectoris (I20) | 42107 | 707 (1.7) | 1682.6 (1392) | 21086 | 393 (1.9) | 1696.4 (1394.3) | 21068 | 383 (1.8) | 1680.7 (1389.2) |
| **Blood vessel** |  |  |  |  |  |  |  |  |  |
| Atherosclerosis (I70) | 46011 | 213 (0.5) | 1701.5 (1390.2) | 23001 | 130 (0.6) | 1715.3 (1393.5) | 23001 | 112 (0.5) | 1700.7 (1386.1) |
| Hypertension (I10) | 29736 | 816 (2.7) | 1713.2 (1398.1) | 14760 | 439 (3.0) | 1724.1 (1396.3) | 14899 | 426 (2.9) | 1698.7 (1391.4) |
| **Brain** |  |  |  |  |  |  |  |  |  |
| Cerebral infarction (I63) | 42267 | 309 (0.7) | 1699 (1395.7) | 21159 | 149 (0.7) | 1717.2 (1398.7) | 21134 | 162 (0.8) | 1699.2 (1393.5) |
| Intracerebral hemorrhage (I61) | 42293 | 77 (0.2) | 1705.1 (1397.6) | 21166 | 43 (0.2) | 1722.8 (1400.3) | 21154 | 37 (0.2) | 1706.1 (1395.6) |
| Subarachnoid hemorrhage (I60) | 42301 | 44 (0.1) | 1705.8 (1397.7) | 21171 | 11 (0.1) | 1724.3 (1400.8) | 21155 | 13 (0.1) | 1707.5 (1396.1) |
| **Metabolism** |  |  |  |  |  |  |  |  |  |
| Type 2 diabetes mellitus (E11) | 22025 | 227 (1.0) | 1753.5 (1392.2) | 11015 | 145 (1.3) | 1770.8 (1393.4) | 11016 | 128 (1.2) | 1741.9 (1381.5) |
| Hyperlipidemia (E78) | 22653 | 492 (2.2) | 1655.1 (1392.9) | 11432 | 268 (2.3) | 1677.5 (1394.5) | 11479 | 236 (2.1) | 1648.1 (1381.7) |
| **Liver** |  |  |  |  |  |  |  |  |  |
| Alcoholic liver disease (K70) | 46062 | 61 (0.1) | 1706.3 (1391.1) | 23033 | 33 (0.1) | 1720.1 (1394.6) | 23037 | 41 (0.2) | 1704.7 (1387.6) |
| Liver fibrosis and cirrhosis (K74) | 46053 | 91 (0.2) | 1705.9 (1390.9) | 23028 | 42 (0.2) | 1719.4 (1394.6) | 23027 | 46 (0.2) | 1705 (1387.7) |
| **Kidney** |  |  |  |  |  |  |  |  |  |
| Chronic kidney disease (N18) | 42239 | 284 (0.7) | 1701.8 (1396.1) | 21148 | 150 (0.7) | 1720.2 (1398.7) | 21138 | 158 (0.7) | 1702.1 (1394.2) |

SD, standard deviation

**Appendix Table 5. Incidence rates in the study population**

| ICD-10 | Disease | Participants, No. | Events,  No. | Person-years | Incidence rate per 10,000 person-years |
| --- | --- | --- | --- | --- | --- |
| I20 | Angina Pectoris | 84261 | 1483 | 388857.9 | 38.1 |
| I21 | Acute Myocardial Infraction | 84577 | 280 | 395618.9 | 7.1 |
| I42 | Cardiomyopathy | 84613 | 99 | 396181.1 | 2.5 |
| I48 | Atrial Fibrillation | 84502 | 835 | 393324.7 | 21.2 |
| I50 | Heart Failure | 84106 | 1858 | 387774.1 | 47.9 |
| I10 | Hypertension | 59395 | 1681 | 278445.1 | 60.4 |
| I70 | Atherosclerosis | 92013 | 455 | 429444.4 | 10.6 |
| I60 | Subarachnoid Hemorrhage | 84627 | 68 | 396385.7 | 1.7 |
| I61 | Intracerebral Hemorrhage | 84613 | 157 | 396088.5 | 4.0 |
| I63 | Cerebral Infarction | 84560 | 620 | 394405.0 | 15.7 |
| E11 | Type 2 Diabetes Mellitus | 44056 | 500 | 211673.9 | 23.6 |
| E78 | Hyperlipidemia | 45564 | 996 | 206943.5 | 48.1 |
| K70 | Alcoholic Liver Disease | 92132 | 135 | 431185.3 | 3.1 |
| K74 | Liver Fibrosis and Cirrhosis | 92108 | 179 | 430972.1 | 4.2 |
| N18 | Chronic Kidney Disease | 84525 | 592 | 394889.7 | 15.0 |

**Appendix Table 6. The follow-up periods** **in the** **two types of groups within event-free group**

| Disease | Type | n | Follow-up duration, median, days | Follow-up duration, min, days | Follow-up duration, max, days | *p* value |
| --- | --- | --- | --- | --- | --- | --- |
| Angina Pectoris | Event-free 1 | 21789 | 689 | 0 | 3920 | <0.001 |
|  | Event-free 2 | 20058 | 2548 | 0 | 3949 |  |
| Acute Myocardial Infraction | Event-free 1 | 21789 | 689 | 0 | 3920 | <0.001 |
|  | Event-free 2 | 20339 | 2557 | 0 | 3949 |  |
| Cardiomyopathy | Event-free 1 | 21789 | 689 | 0 | 3920 | <0.001 |
|  | Event-free 2 | 19315 | 2542 | 0 | 3947 |  |
| Atrial Fibrillation | Event-free 1 | 21789 | 689 | 0 | 3920 | <0.001 |
|  | Event-free 2 | 20455 | 2556 | 0 | 3947 |  |
| Heart Failure | Event-free 1 | 21789 | 689 | 0 | 3920 | <0.001 |
|  | Event-free 2 | 19611 | 2547 | 0 | 3947 |  |
| Hypertension | Event-free 1 | 22945 | 679 | 0 | 3920 | <0.001 |
|  | Event-free 2 | 22853 | 2508 | 0 | 3949 |  |
| Atherosclerosis | Event-free 1 | 16003 | 714 | 0 | 3920 | <0.001 |
|  | Event-free 2 | 12917 | 2630 | 0 | 3947 |  |
| Subarachnoid Hemorrhage | Event-free 1 | 21789 | 689 | 0 | 3920 | <0.001 |
|  | Event-free 2 | 20169 | 2553 | 0 | 3949 |  |
| Intracerebral Hemorrhage | Event-free 1 | 21789 | 689 | 0 | 3920 | <0.001 |
|  | Event-free 2 | 20427 | 2557 | 0 | 3949 |  |
| Cerebral Infarction | Event-free 1 | 21789 | 689 | 0 | 3920 | <0.001 |
|  | Event-free 2 | 20468 | 2556 | 0 | 3949 |  |
| Type 2 Diabetes Mellitus | Event-free 1 | 11092 | 700 | 0 | 3827 | <0.001 |
|  | Event-free 2 | 10706 | 2651 | 0 | 3859 |  |
| Hyperlipidemia | Event-free 1 | 12152 | 653 | 0 | 3915 | <0.001 |
|  | Event-free 2 | 10009 | 2548 | 0 | 3942 |  |
| Alcoholic Liver Disease | Event-free 1 | 22945 | 679 | 0 | 3920 | <0.001 |
|  | Event-free 2 | 23056 | 2513 | 0 | 3949 |  |
| Liver Fibrosis and Cirrhosis | Event-free 1 | 22945 | 679 | 0 | 3920 | <0.001 |
|  | Event-free 2 | 23017 | 2512 | 0 | 3949 |  |
| Chronic Kidney Disease | Event-free 1 | 21789 | 689 | 0 | 3920 | <0.001 |
|  | Event-free 2 | 20166 | 2553 | 0 | 3949 |  |

*p* values of Wilcoxon rank sum test. Bonferroni-corrected P-values are reported. Event-free 1: the participants in this group did not have any diagnosis during the period from the first visit for a health checkup to their last visit for a health checkup. Event-free 2: the event-free group that follow-up period was defined by their last visit for a health checkup or the latest diagnosis of other diseases, whichever was later.

**Appendix Table 7. Risk prediction models for heart diseases**

|  | **β (standard error)** | **HR** | **z** | ***P* value** |
| --- | --- | --- | --- | --- |
| **Angina pectoris** |  |  |  |  |
| Age | 8.5369 (0.5796) | 5099.5308 | 14.729 | <0.001 |
| GLU | 1.33817 (0.6993) | 3.81207 | 1.914 | 0.055674 |
| Height | 8.85341 (2.08384) | 6998.2253 | 4.249 | <0.001 |
| Systolic blood pressure | 4.88944 (1.27781) | 132.879 | 3.826 | <0.001 |
| Diastolic blood pressure | -3.04731 (1.28587) | 0.04749 | -2.37 | 0.017796 |
| Diabetes mellitus | 0.58522 (0.16516) | 1.79538 | 3.543 | <0.001 |
| Hypertension | 0.19229 (0.0991) | 1.21203 | 1.94 | 0.052326 |
| Smoking | 0.2516 (0.10887) | 1.28608 | 2.311 | 0.020836 |
| Hyperlipidemia | 0.13757 (0.1104) | 1.14748 | 1.246 | 0.212719 |
| Weight gain from age 20 years | -0.10717 (0.08951) | 0.89837 | -1.197 | 0.231175 |
| TG | 0.21757 (0.21086) | 1.24305 | 1.032 | 0.302167 |
| γ-GTP | -0.29784 (0.15457) | 0.74242 | -1.927 | 0.053987 |
| UA | 1.47694 (0.43391) | 4.37951 | 3.404 | <0.001 |
| Walking speed | -0.16248 (0.08237) | 0.85004 | -1.972 | 0.048555 |
| **Acute myocardial infarction** |  |  |  |  |
| Age | 5.39213 (1.18601) | 219.6702 | 4.546 | <0.001 |
| Systolic blood pressure | 6.06287 (1.58621) | 429.60545 | 3.822 | <0.001 |
| Diabetes mellitus | 0.67748 (0.28783) | 1.9689 | 2.354 | 0.018586 |
| Weight change in past 1 year | 0.3465 (0.19732) | 1.41411 | 1.756 | 0.079083 |
| Walking speed | -0.55434 (0.19047) | 0.57445 | -2.91 | 0.00361 |
| Men | 1.09323 (0.24008) | 2.9839 | 4.554 | <0.001 |
| UA | -1.19838 (0.86265) | 0.30168 | -1.389 | 0.164779 |
| γ-GTP | -0.72874 (0.378) | 0.48251 | -1.928 | 0.053868 |
| AST | 3.38748 (0.97343) | 29.59129 | 3.48 | <0.001 |
| LDL | 1.82704 (0.8641) | 6.21547 | 2.114 | 0.034483 |
| Hyperlipidemia | 0.47288 (0.23061) | 1.6046 | 2.051 | 0.040312 |
| ALT | -1.44824 (0.67138) | 0.23498 | -2.157 | 0.030998 |
| HDL | -2.9675 (0.94938) | 0.05143 | -3.126 | 0.001774 |
| **Cardiomyopathy** |  |  |  |  |
| Age | 10.62 (2.097) | 40930 | 5.064 | <0.001 |
| Diastolic blood pressure | 9.494 (2.729) | 13280 | 3.48 | <0.001 |
| Skipping breakfast | 0.912 (0.4284) | 2.489 | 2.129 | 0.033268 |
| Weight gain from age 20 years | -0.5323 (0.3338) | 0.5872 | -1.595 | 0.110786 |
| HDL | -3.855 (1.448) | 0.02118 | -2.662 | 0.007758 |
| Eating snacks | -0.0292 (0.4427) | 0.9712 | -0.066 | 0.947414 |
| **Atrial fibrillation and flutter** |  |  |  |  |
| Age | 13.36 (0.818) | 635200 | 16.335 | <0.001 |
| Height | 22.45 (2.473) | 5.649E+09 | 9.08 | <0.001 |
| Diastolic blood pressure | 3.266 (0.9705) | 26.21 | 3.365 | <0.001 |
| Walking speed | -0.2994 (0.108) | 0.7412 | -2.774 | 0.005545 |
| Hypertension | 0.3894 (0.1178) | 1.476 | 3.307 | <0.001 |
| Diabetes mellitus | -0.006744 (0.218) | 0.9933 | -0.031 | 0.975326 |
| **Heart failure** |  |  |  |  |
| Age | 8.767 (0.53352) | 6418.8591 | 16.432 | <0.001 |
| Systolic blood pressure | 3.10228 (0.64) | 22.24862 | 4.847 | <0.001 |
| Hypertension | 0.35114 (0.0819) | 1.42068 | 4.287 | <0.001 |
| Weight change in 1 year | 0.31352 (0.08141) | 1.36823 | 3.851 | <0.001 |
| Sleeping | -0.2273 (0.07417) | 0.79668 | -3.065 | 0.002178 |
| HDL | -1.22023 (0.37714) | 0.29516 | -3.235 | 0.001214 |
| Walking speed | -0.1514 (0.07175) | 0.8595 | -2.11 | 0.034837 |
| UA | 0.89285 (0.36593) | 2.44208 | 2.44 | 0.014689 |
| Diabetes mellitus | 0.19288 (0.1412) | 1.21274 | 1.366 | 0.171927 |
| ALT | -1.06026 (0.27592) | 0.34637 | -3.843 | <0.001 |
| AST | 1.3298 (0.41642) | 3.78029 | 3.193 | 0.001406 |
| Weight | 1.56688 (0.58039) | 4.79169 | 2.7 | 0.00694 |
| Eating late dinner | -0.10991 (0.09226) | 0.89592 | -1.191 | 0.233568 |

HR, hazard ratio; ALT, alanine transaminase; AST, aspartate transaminase; γ-GTP, γ-glutamyl transpeptidase; GLU, fasting plasma glucose; HDL, high-density lipoprotein cholesterol; LDL, low-density lipoprotein cholesterol; TG, triglyceride; UA, uric acid

Continuous variables were converted to log space with base 10 before analysis.

**Appendix Table 8. Risk prediction models for blood vessel diseases**

|  | **β (standard error)** | **HR** |  | **z** | ***P* value** |
| --- | --- | --- | --- | --- | --- |
| **Hypertension** |  |  |  |  |  |
| Age | 6.79665 (0.5613) | 894.84339 |  | 12.109 | <0.001 |
| Systolic blood pressure | 5.07015 (1.86808) | 159.199 |  | 2.714 | 0.00665 |
| Diabetes mellitus | 0.78553 (0.17297) | 2.19357 |  | 4.541 | <0.001 |
| WC | 2.85332 (1.07402) | 17.34526 |  | 2.657 | 0.00789 |
| Diastolic blood pressure | 4.49206 (1.81541) | 89.30523 |  | 2.474 | 0.01335 |
| Drinking volume 180 - 360 mL | 0.15366 (0.10395) | 1.16609 |  | 1.478 | 0.13935 |
| Drinking volume 360 - 540 mL | 0.14836 (0.14566) | 1.15993 |  | 1.019 | 0.30843 |
| Drinking volume more than 540 mL | 0.58776 (0.22097) | 1.79995 |  | 2.66 | 0.00782 |
| Walking speed | -0.17115 (0.09283) | 0.84269 |  | -1.844 | 0.06522 |
| **Atherosclerosis** |  |  |  |  |  |
| Age | 10.96 (1.161) | 57760 |  | 9.446 | <0.001 |
| GLU | 4.256 (0.9571) | 70.56 |  | 4.447 | <0.001 |
| Smoking | 0.7965 (0.1823) | 2.218 |  | 4.369 | <0.001 |
| UA | 1.888 (0.7415) | 6.608 |  | 2.546 | 0.01088 |
| Hyperlipidemia | 0.3057 (0.1774) | 1.358 |  | 1.723 | 0.08487 |
| Diabetes mellitus | 0.6821 (0.2485) | 1.978 |  | 2.745 | 0.00605 |
| Systolic blood pressure | 3.871 (2.022) | 48 |  | 1.914 | 0.05558 |
| Diastolic blood pressure | -2.867 (1.925) | 0.05686 |  | -1.489 | 0.13638 |

HR, hazard ratio; GLU, fasting plasma glucose; UA, uric acid; WC, waist circumference

Continuous variables were converted to log space with base 10 before analysis

**Appendix Table 9. Risk prediction models for brain diseases**

|  | **β (standard error)** | **HR** | **z** | ***P* value** |
| --- | --- | --- | --- | --- |
| **Subarachnoid hemorrhage** |  |  |  |  |
| Weight | -7.455 (2.112) | 0.0005786 | -3.53 | <0.001 |
| Systolic blood pressure | 11.1 (2.609) | 66320 | 4.255 | <0.001 |
| GLU | -5.23 (3.593) | 0.005351 | -1.456 | 0.14551 |
| Walking speed | -0.1072 (0.3233) | 0.8984 | -0.332 | 0.740221 |
| **Intracerebral hemorrhage** |  |  |  |  |
| Diastolic blood pressure | 14.21 (2.287) | 1487000 | 6.214 | <0.001 |
| Age | 5.553 (1.584) | 258 | 3.505 | <0.001 |
| BMI | -10.35 (2.161) | 3.208E-05 | -4.789 | <0.001 |
| Walking speed | -0.6738 (0.2647) | 0.5098 | -2.545 | 0.010913 |
| Diabetes mellitus | 0.5479 (0.628) | 1.73 | 0.873 | 0.382923 |
| GLU | 0.3084 (2.21) | 1.361 | 0.14 | 0.888992 |
| γ-GTP | 0.4789 (0.3657) | 1.614 | 1.309 | 0.190385 |
| **Cerebral infarction** |  |  |  |  |
| Age | 10.4 (0.9056) | 32870 | 11.484 | <0.001 |
| Diabetes mellitus | 1.234 (0.2262) | 3.434 | 5.455 | <0.001 |
| Systolic blood pressure | 5.03 (1.098) | 152.9 | 4.581 | <0.001 |
| UA | 2.11 (0.6117) | 8.245 | 3.449 | <0.001 |
| ALT | -0.9693 (0.3335) | 0.3794 | -2.906 | 0.00366 |
| Smoking | 0.487 (0.1607) | 1.627 | 3.03 | 0.002442 |
| GLU | 0.04508 (1.075) | 1.046 | 0.042 | 0.966554 |

HR, hazard ratio; ALT, alanine transaminase; BMI, body mass index; γ-GTP, γ-glutamyl transpeptidase; GLU, fasting plasma glucose; UA, uric acid

Continuous variables were converted into log space with base 10 before analysis

**Appendix Table 10. Risk prediction models for metabolic diseases**

|  | **β (standard error)** | **HR** | **z** | ***P* value** |
| --- | --- | --- | --- | --- |
| **Type 2 diabetes mellitus** |  |  |  |  |
| HbA1c | 26.26 (4.131) | 2.531E+11 | 6.355 | <0.001 |
| AST | 3.776 (0.8162) | 43.66 | 4.627 | <0.001 |
| WC | 4.764 (1.917) | 117.3 | 2.485 | 0.01294 |
| Age | 3.708 (1.169) | 40.76 | 3.173 | 0.00151 |
| TG | 0.6881 (0.406) | 1.99 | 1.695 | 0.09008 |
| Walking speed | -0.3774 (0.1726) | 0.6856 | -2.187 | 0.02872 |
| Drinking volume 180 - 360 mL | -0.278 (0.2235) | 0.7573 | -1.244 | 0.21358 |
| Drinking volume 360 - 540 mL | -0.09736 (0.2735) | 0.9072 | -0.356 | 0.72191 |
| Drinking volume more than 540 mL | 0.7473 (0.3322) | 2.111 | 2.25 | 0.02448 |
| Drinking frequency–Sometimes | -0.5148 (0.2235) | 0.5976 | -2.304 | 0.02125 |
| Drinking frequency–Everyday | -0.4917 (0.2533) | 0.6116 | -1.941 | 0.05221 |
| Eating late dinner | 0.3263 (0.1819) | 1.386 | 1.794 | 0.07279 |
| Hypertension | 0.2905 (0.1915) | 1.337 | 1.518 | 0.12913 |
| Skipping breakfast | 0.4315 (0.2411) | 1.54 | 1.79 | 0.07348 |
| HDL | -1.735 (0.9527) | 0.1764 | -1.821 | 0.06862 |
| ALT | -1.187 (0.6174) | 0.3052 | -1.922 | 0.05459 |
| GLU | 3.27 (2.476) | 26.32 | 1.321 | 0.18664 |
| **Hyperlipidemia** |  |  |  |  |
| Age | 5.2293 (0.5711) | 186.6566 | 9.157 | <0.001 |
| LDL | 2.8686 (0.6369) | 17.6119 | 4.504 | <0.001 |
| GLU | 1.3393 (0.8676) | 3.8163 | 1.544 | 0.122661 |
| AST | 1.5639 (0.3177) | 4.7774 | 4.922 | <0.001 |
| Weight change in past 1 year | 0.3503 (0.1041) | 1.4196 | 3.366 | <0.001 |
| Eating snacks | 0.1865 (0.1255) | 1.205 | 1.486 | 0.137251 |
| Diabetes mellitus | 0.8642 (0.22) | 2.3732 | 3.928 | <0.001 |
| TG | 0.8067 (0.308) | 2.2406 | 2.619 | 0.008809 |
| Eating late dinner | -0.1858 (0.1192) | 0.8304 | -1.559 | 0.118901 |
| Exercise | 0.1352 (0.1021) | 1.1448 | 1.325 | 0.185114 |

HR, hazard ratio; ALT, alanine transaminase; AST, aspartate transaminase; GLU, fasting plasma glucose; HbA1c, hemoglobin A1c; HDL, high-density lipoprotein cholesterol; LDL, low-density lipoprotein cholesterol; TG, triglyceride; WC, waist circumference

Continuous variables were converted to log space with base 10 before analysis

**Appendix Table 11. Risk prediction models for liver diseases**

|  | **β (standard error)** | **HR** | **z** | ***P* value** |
| --- | --- | --- | --- | --- |
| **Alcoholic liver disease** |  |  |  |  |
| γ-GTP | 2.3374 (0.4077) | 10.3541 | 5.734 | <0.001 |
| Drinking volume 180 - 360 mL | 1.1863 (0.5738) | 3.2749 | 2.067 | 0.038694 |
| Drinking volume 360 - 540 mL | 2.1652 (0.5629) | 8.7167 | 3.846 | <0.001 |
| Drinking volume more than 540 mL | 1.8402 (0.6706) | 6.2975 | 2.744 | 0.006069 |
| Age | 4.7961 (2.0638) | 121.0329 | 2.324 | 0.02013 |
| Diabetes mellitus | 1.1218 (0.3332) | 3.0704 | 3.367 | <0.001 |
| AST | 3.6277 (0.9646) | 37.6256 | 3.761 | <0.001 |
| ALT | -1.8958 (0.8993) | 0.1502 | -2.108 | 0.035018 |
| Hypertension | 0.5616 (0.2967) | 1.7534 | 1.892 | 0.058432 |
| **Fibrosis and cirrhosis of liver** |  |  |  |  |
| AST | 5.833 (0.8902) | 341.3 | 6.552 | <0.001 |
| HDL | -4.352 (1.093) | 0.01289 | -3.98 | <0.001 |
| Height | -19.02 (5.425) | 5.474E-09 | -3.507 | <0.001 |
| Hypertension | -0.3831 (0.3106) | 0.6817 | -1.233 | 0.217428 |
| Exercise | 0.4937 (0.2506) | 1.638 | 1.97 | 0.048813 |
| WC | 5.456 (2.743) | 234.1 | 1.989 | 0.046745 |
| ALT | -2.258 (0.7841) | 0.1046 | -2.88 | 0.003982 |
| γ-GTP | 1.759 (0.3617) | 5.805 | 4.863 | <0.001 |
| LDL | 1.331 (1.018) | 3.785 | 1.307 | 0.191152 |
| Diastolic blood pressure | -5.567 (1.961) | 0.003822 | -2.839 | 0.00452 |

HR, hazard ratio; ALT, alanine transaminase; AST, aspartate transaminase; γ -GTP, γ -glutamyl transpeptidase; HDL, high-density lipoprotein cholesterol; LDL, low-density lipoprotein cholesterol; WC, waist circumference

Continuous variables were converted to log space with base 10 before analysis

**Appendix Table 12. Risk prediction models for chronic kidney disease**

|  | **β (standard error)** | **HR** | **z** | ***P* value** |
| --- | --- | --- | --- | --- |
| **Chronic kidney disease** |  |  |  |  |
| Age | 6.8252 (0.8827) | 920.7231 | 7.732 | <0.001 |
| UA | 5.4558 (0.6913) | 234.1156 | 7.892 | <0.001 |
| ALT | -1.8544 (0.4879) | 0.1566 | -3.8 | <0.001 |
| AST | 2.4991 (0.7522) | 12.1713 | 3.322 | <0.001 |
| Diabetes mellitus | 1.1207 (0.2644) | 3.067 | 4.239 | <0.001 |
| γ-GTP | -0.6525 (0.2744) | 0.5208 | -2.378 | 0.017418 |
| Systolic blood pressure | 3.2771 (1.1402) | 26.4986 | 2.874 | 0.004051 |
| GLU | -0.9208 (1.2877) | 0.3982 | -0.715 | 0.474564 |

HR, hazard ratio; ALT, alanine transaminase; AST, aspartate transaminase; AST, aspartate transaminase; γ-GTP, γ-glutamyl transpeptidase; GLU, fasting plasma glucose; UA, uric acid

Continuous variables were converted to log space with base 10 before analysis

**Appendix Table 13. C-index for the risk prediction models**

|  |  | **C-index** |  |
| --- | --- | --- | --- |
|  | **Derivation** | **Verification** | **Validation** |
| **Heart** |  |  |  |
| Atrial fibrillation (I48) | 0.82 | 0.806 | 0.813 |
| Acute myocardial infraction (I21) | 0.772 | 0.753 | 0.82 |
| Heart failure (I50) | 0.75 | 0.738 | 0.763 |
| Cardiomyopathy (I42) | 0.835 | 0.861 | 0.723 |
| Angina pectoris (I20) | 0.744 | 0.704 | 0.708 |
| **Blood Vessel** |  |  |  |
| Atherosclerosis (I70) | 0.822 | 0.767 | 0.803 |
| Hypertension (I10) | 0.747 | 0.753 | 0.775 |
| **Brain** |  |  |  |
| Cerebral infarction (I63) | 0.785 | 0.778 | 0.783 |
| Intracerebral hemorrhage (I61) | 0.795 | 0.743 | 0.684 |
| Subarachnoid hemorrhage (I60) | 0.728 | 0.774 | 0.551 |
| **Metabolism** |  |  |  |
| Type 2 diabetes mellitus (E11) | 0.828 | 0.79 | 0.791 |
| Hyperlipidemia (E78) | 0.731 | 0.708 | 0.699 |
| **Liver** |  |  |  |
| Alcoholic liver disease (K70) | 0.954 | 0.916 | 0.898 |
| Liver fibrosis and cirrhosis (K74) | 0.796 | 0.76 | 0.842 |
| **Kidney** |  |  |  |
| Chronic kidney disease (N18) | 0.799 | 0.744 | 0.762 |

**Appendix Table 14. Calibration of risk prediction models using Hosmer-Lemeshow test**

|  |  | ***P* value** |  |
| --- | --- | --- | --- |
|  | **Derivation** | **Verification** | **Validation** |
| **Heart** |  |  |  |
| Atrial fibrillation (I48) | 0.039 | 0.003 | 0.037 |
| Acute myocardial infraction (I21) | 0.244 | 0.075 | 0.615 |
| Heart failure (I50) | 0.17 | <0.001 | 0.001 |
| Cardiomyopathy (I42) | 0.35 | 0.773 | 0.098 |
| Angina pectoris (I20) | 0.021 | <0.001 | <0.001 |
| **Blood Vessel** |  |  |  |
| Atherosclerosis (I70) | 0.001 | 0.059 | 0.581 |
| Hypertension (I10) | 0.183 | 0.031 | 0.003 |
| **Brain** |  |  |  |
| Cerebral infarction (I63) | 0.149 | 0.059 | 0.186 |
| Intracerebral hemorrhage (I61) | 0.367 | 0.231 | 0.493 |
| Subarachnoid hemorrhage (I60) | 0.205 | 0.641 | 0.052 |
| **Metabolism** |  |  |  |
| Type 2 diabetes mellitus (E11) | 0.102 | 0.019 | 0.115 |
| Hyperlipidemia (E78) | 0.071 | 0.003 | 0.846 |
| **Liver** |  |  |  |
| Alcoholic liver disease (K70) | 0.844 | 0.826 | 0.002 |
| Liver fibrosis and cirrhosis (K74) | 0.014 | 0.191 | 0.28 |
| **Kidney** |  |  |  |
| Chronic kidney disease (N18) | 0.197 | 0.085 | 0.215 |


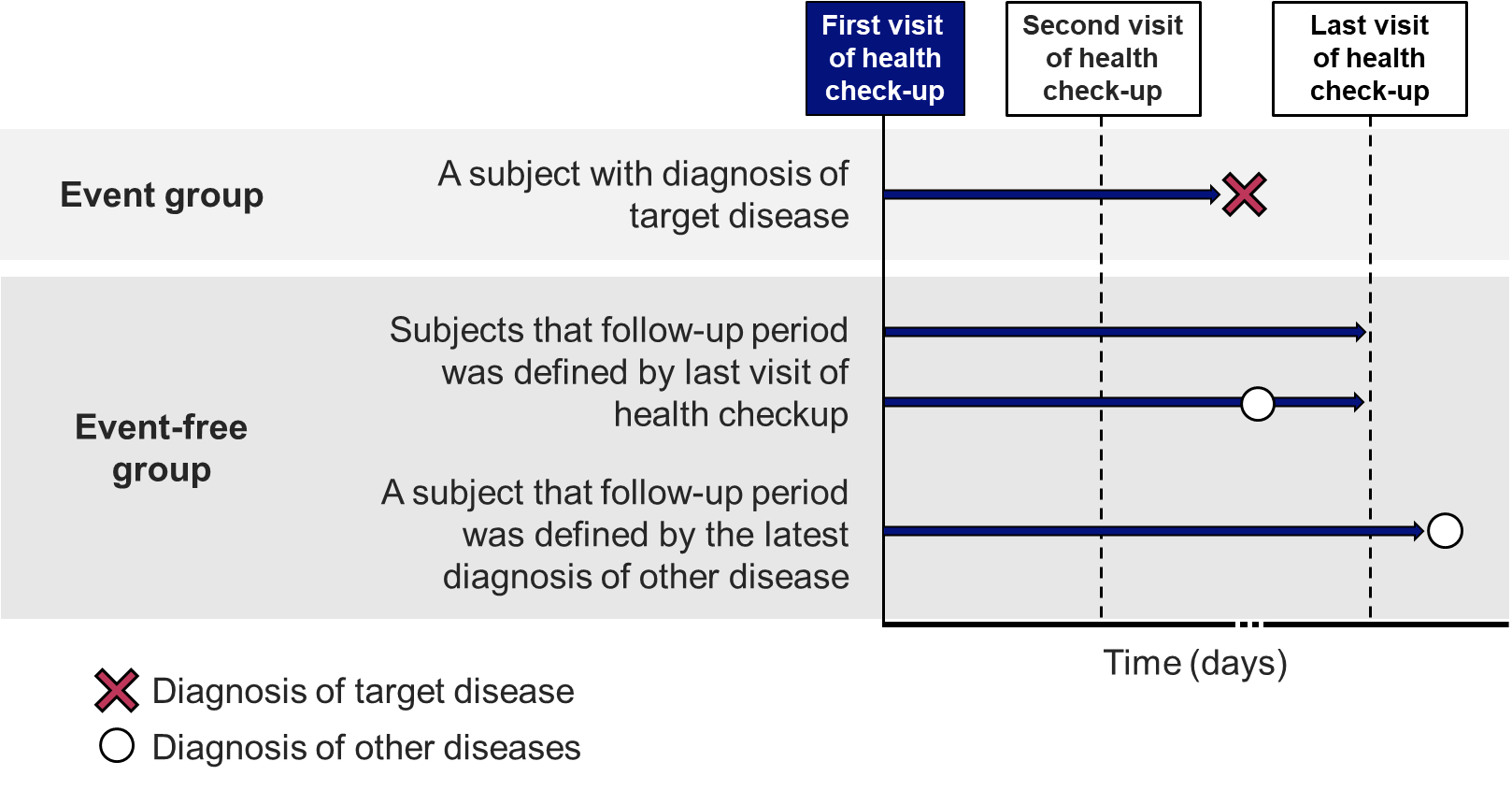


**Appendix Figure 1. The schematic describing typical timeline for survival analysis**

The follow-up began at the time of each participant's first visit for health checkup. When participants had no events (event-free group), the survival time was defined as the period from the first visit for a health checkup to their last visit for a health checkup or the latest diagnosis of other diseases, whichever was later.

**
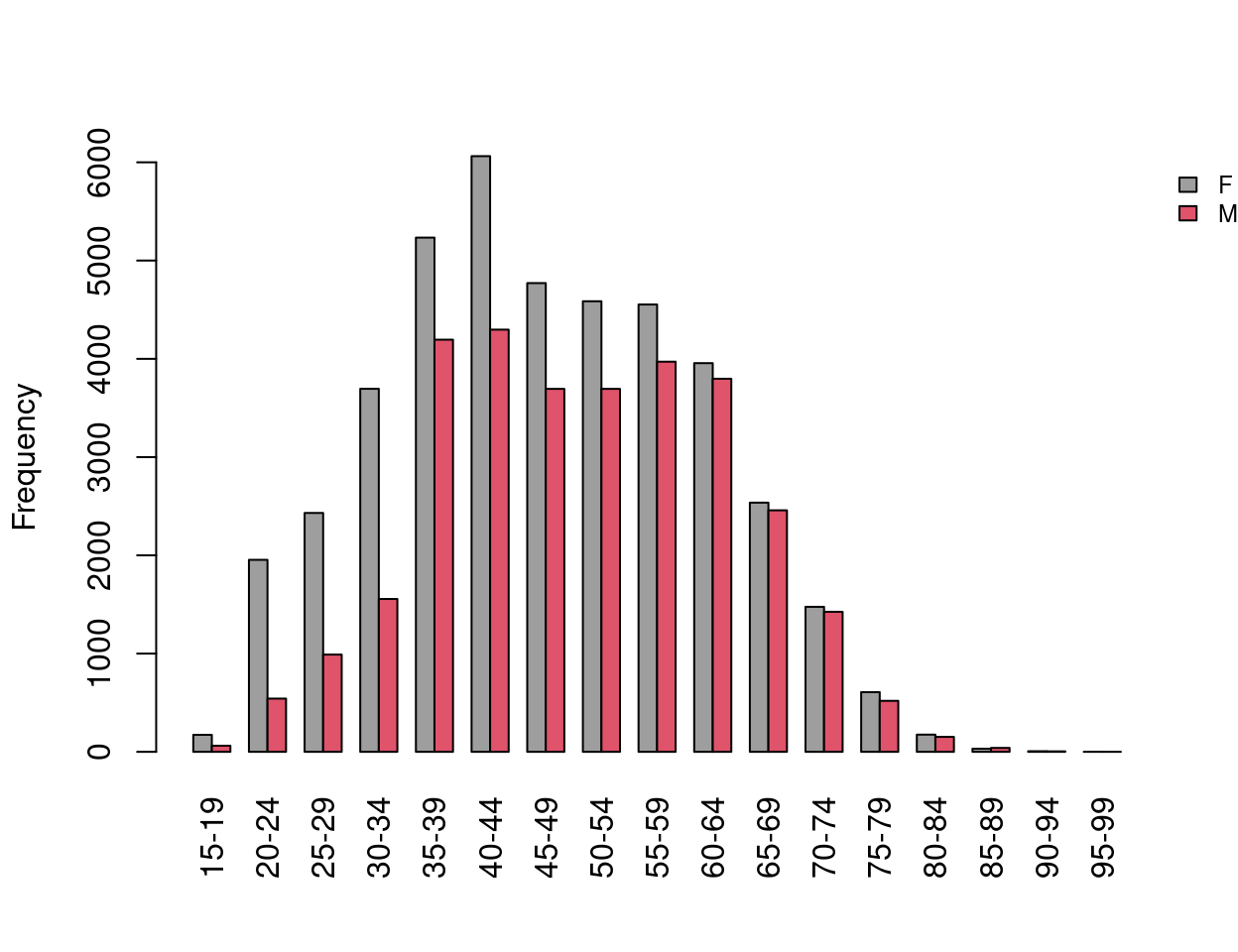
** **Appendix Figure 2. The age distribution of participants (n = 92 174)**

**
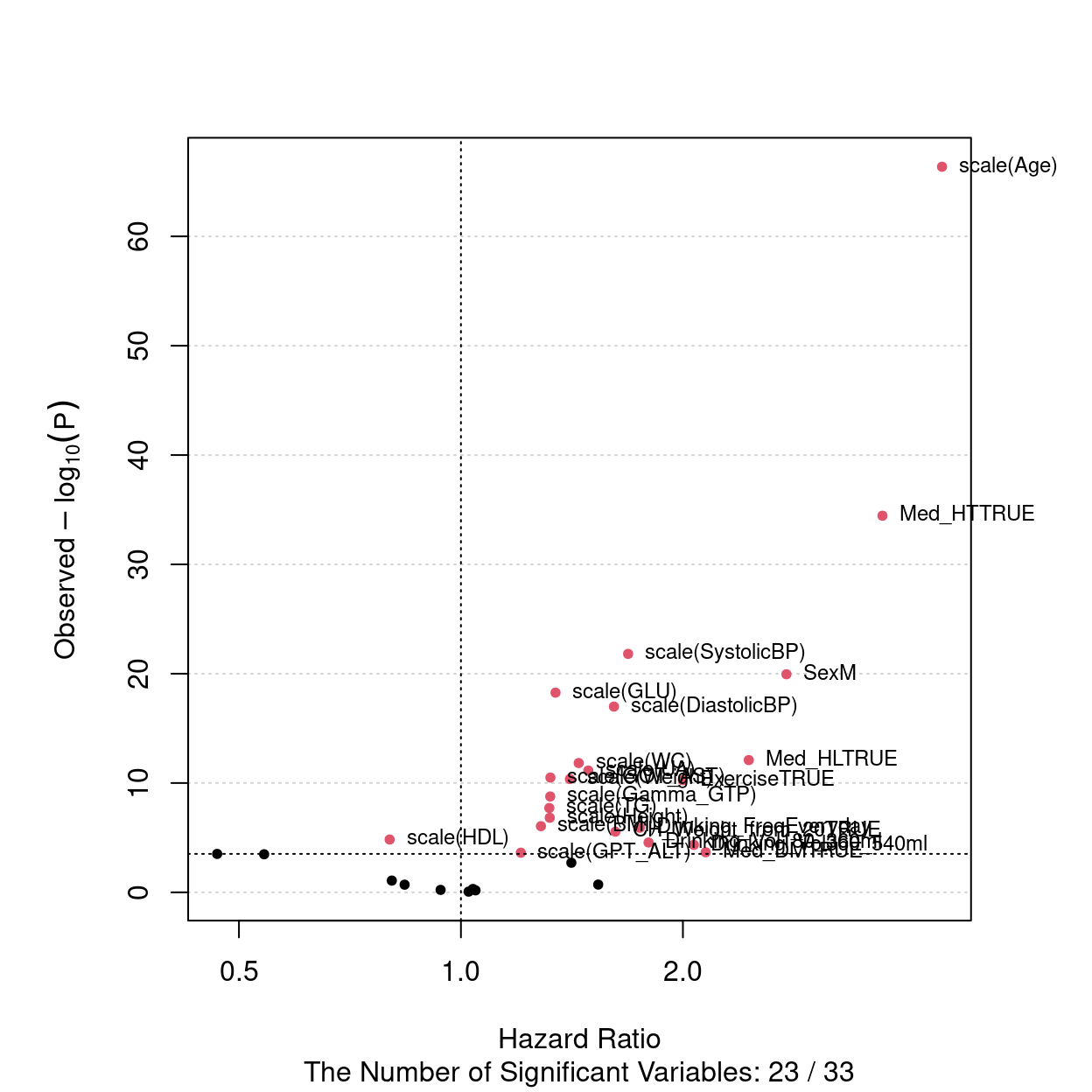
 Appendix Figure 3. Associations between exposure factors and atrial fibrillation**

Cox proportional hazards model revealed the exposure factors associated with a particular event at a 1% Bonferroni-corrected level. Hazard ratios and log_10_(P) values were plotted on a volcanic plot. Continuous variables were converted to log space with base 10 before analysis. Continuous variables were scaled such that hazard ratios reflected incremental changes in hazard per 1 standard deviation change in the predictor.

ALT, alanine transaminase; AST, aspartate transaminase; BMI, body mass index; BP, blood pressure; DM, diabetes mellitus; γ-GTP, γ-glutamyl transpeptidase; GLU, fasting plasma glucose; HDL, high-density lipoprotein cholesterol; HT, hypertension; HL, hyperlipidemia; DM, diabetes mellitus; TG, triglyceride; UA, uric acid; WC, waist circumference

**
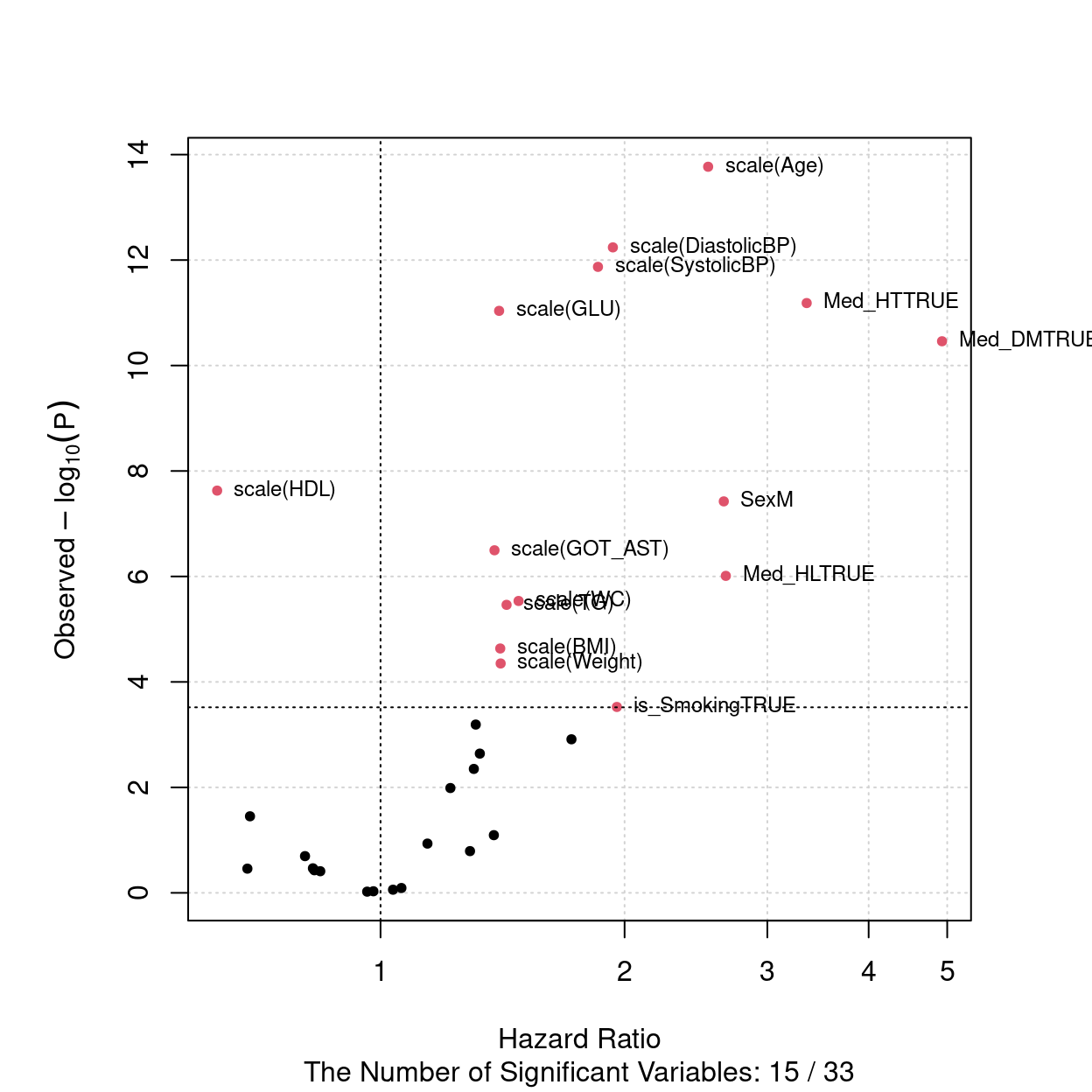
 Appendix Figure 4. Associations between exposure factors and acute myocardial infraction**

Cox proportional hazards model revealed the exposure factors associated with a particular event at a 1% Bonferroni-corrected level. Hazard ratios and log_10_(P) values were plotted on a volcanic plot. Continuous variables were converted to log space with base 10 before analysis. Continuous variables were scaled such that hazard ratios reflected incremental changes in hazard per 1 standard deviation change in the predictor.

AST, aspartate transaminase; BMI, body mass index; BP, blood pressure; DM, diabetes mellitus; GLU, fasting plasma glucose; HDL, high-density lipoprotein cholesterol; HT, hypertension; HL, hyperlipidemia; TG, triglyceride; WC, waist circumference; M, men

**
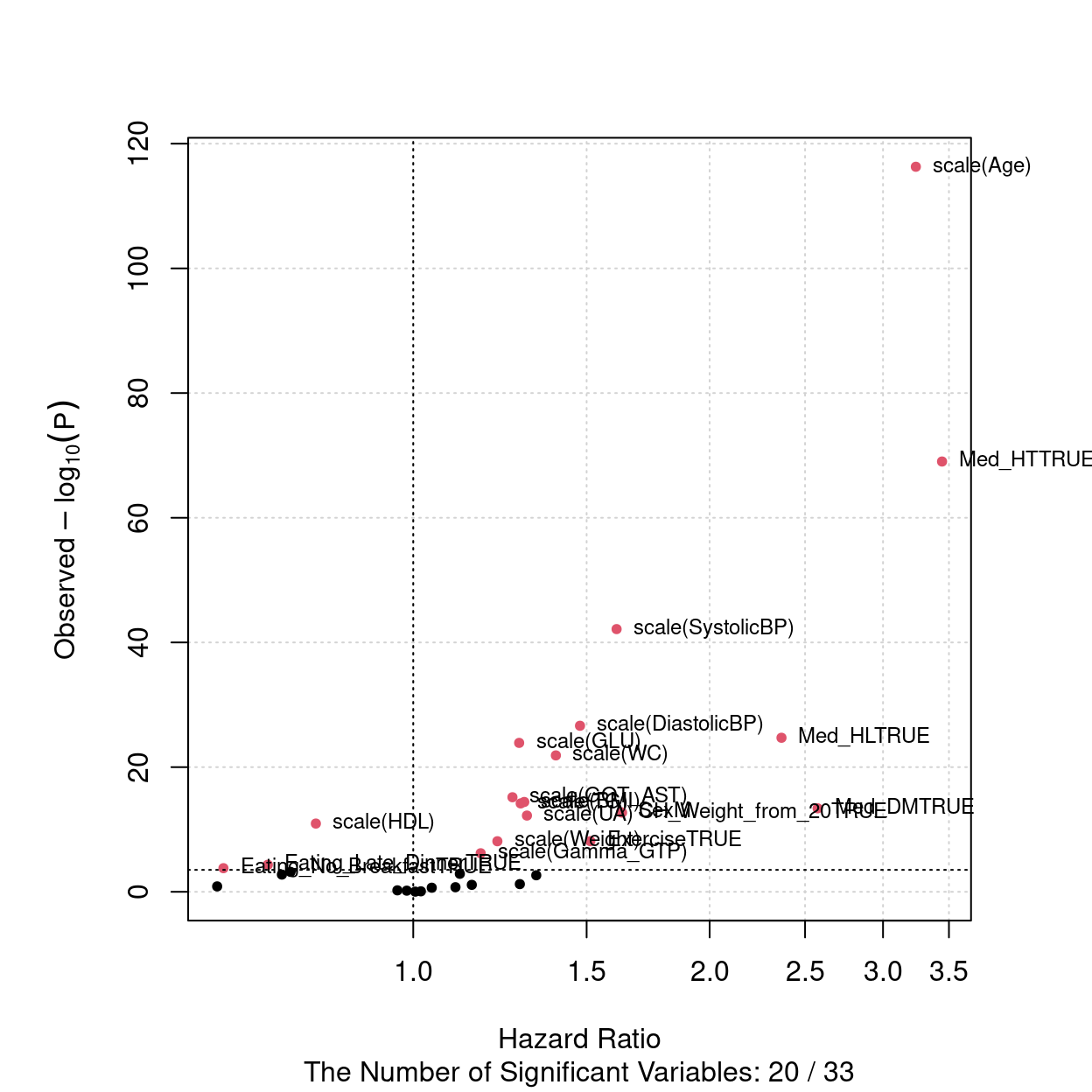
 Appendix Figure 5. Associations between exposure factors and heart failure**

Cox proportional hazards model revealed the exposure factors associated with a particular event at a 1% Bonferroni-corrected level. Hazard ratios and log_10_(P) values were plotted on a volcanic plot. Continuous variables were converted to log space with base 10 before analysis. Continuous variables were scaled such that hazard ratios reflected incremental changes in hazard per 1 standard deviation change in the predictor.

AST, aspartate transaminase; BMI, body mass index; BP, blood pressure; DM, diabetes mellitus; γ-GTP, γ-glutamyl transpeptidase; GLU, fasting plasma glucose; HDL, high-density lipoprotein cholesterol; HT, hypertension; HL, hyperlipidemia; TG, triglyceride; UA, uric acid; WC, waist circumference
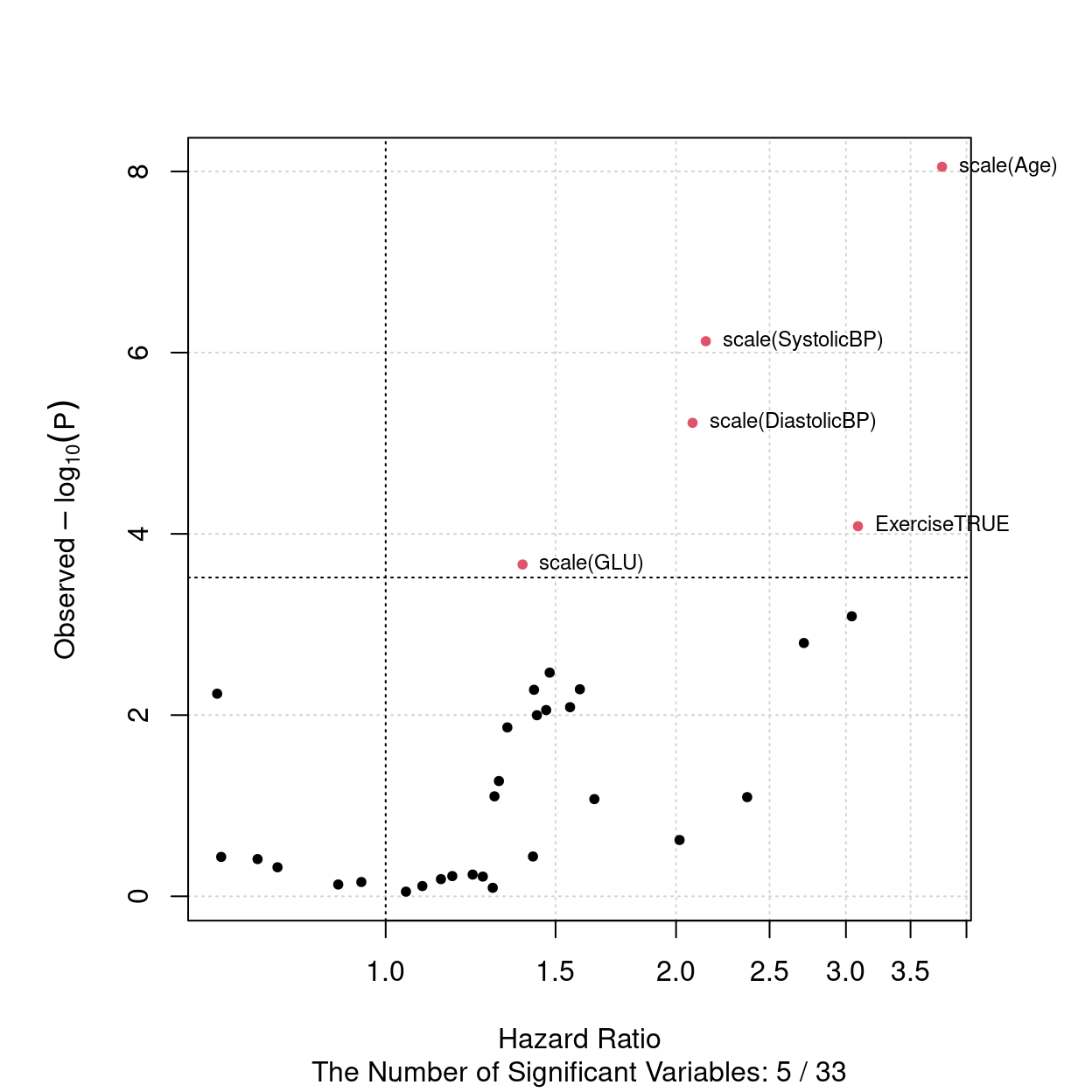
 **Appendix Figure 6. Associations between exposure factors and cardiomyopathy**

Cox proportional hazards model revealed the exposure factors associated with a particular event at a 1% Bonferroni-corrected level. Hazard ratios and log_10_(P) values were plotted on a volcanic plot. Continuous variables were converted to log space with base 10 before analysis. Continuous variables were scaled such that hazard ratios reflected incremental changes in hazard per 1 standard deviation change in the predictor.

BP, blood pressure; GLU, fasting plasma glucose

**
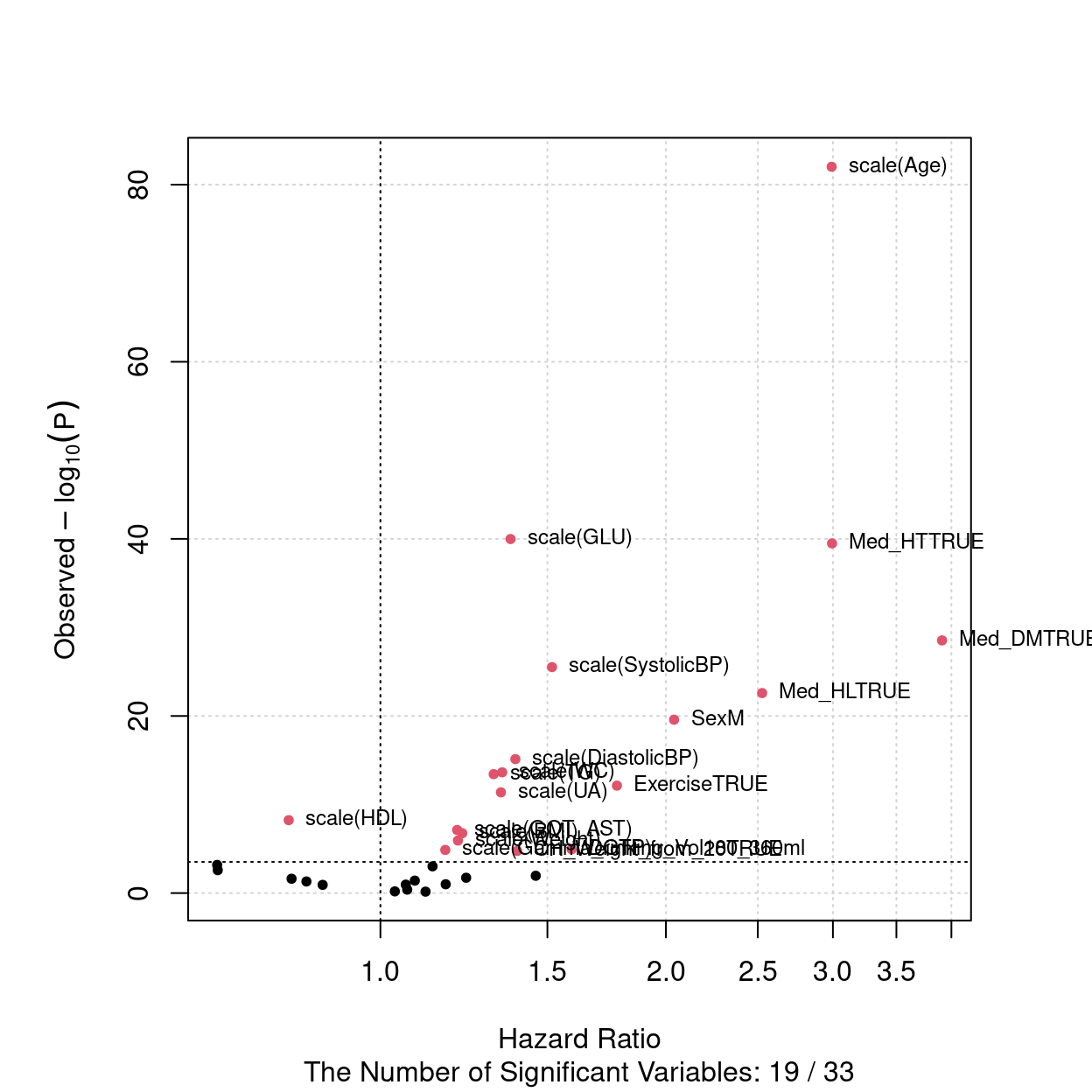
 Appendix Figure 7. Associations between exposure factors and angina pectoris**

Cox proportional hazards model revealed the exposure factors associated with a particular event at a 1% Bonferroni-corrected level. Hazard ratios and log_10_(P) values were plotted on a volcanic plot. Continuous variables were converted to log space with base 10 before analysis. Continuous variables were scaled such that hazard ratios reflected incremental changes in hazard per 1 standard deviation change in the predictor.

AST, aspartate transaminase; BMI, body mass index; BP, blood pressure; DM, diabetes mellitus; γ-GTP, γ-glutamyl transpeptidase; GLU, fasting plasma glucose; HDL, high-density lipoprotein cholesterol; HT, hypertension; HL, hyperlipidemia; TG, triglyceride; UA, uric acid; WC, waist circumference

**
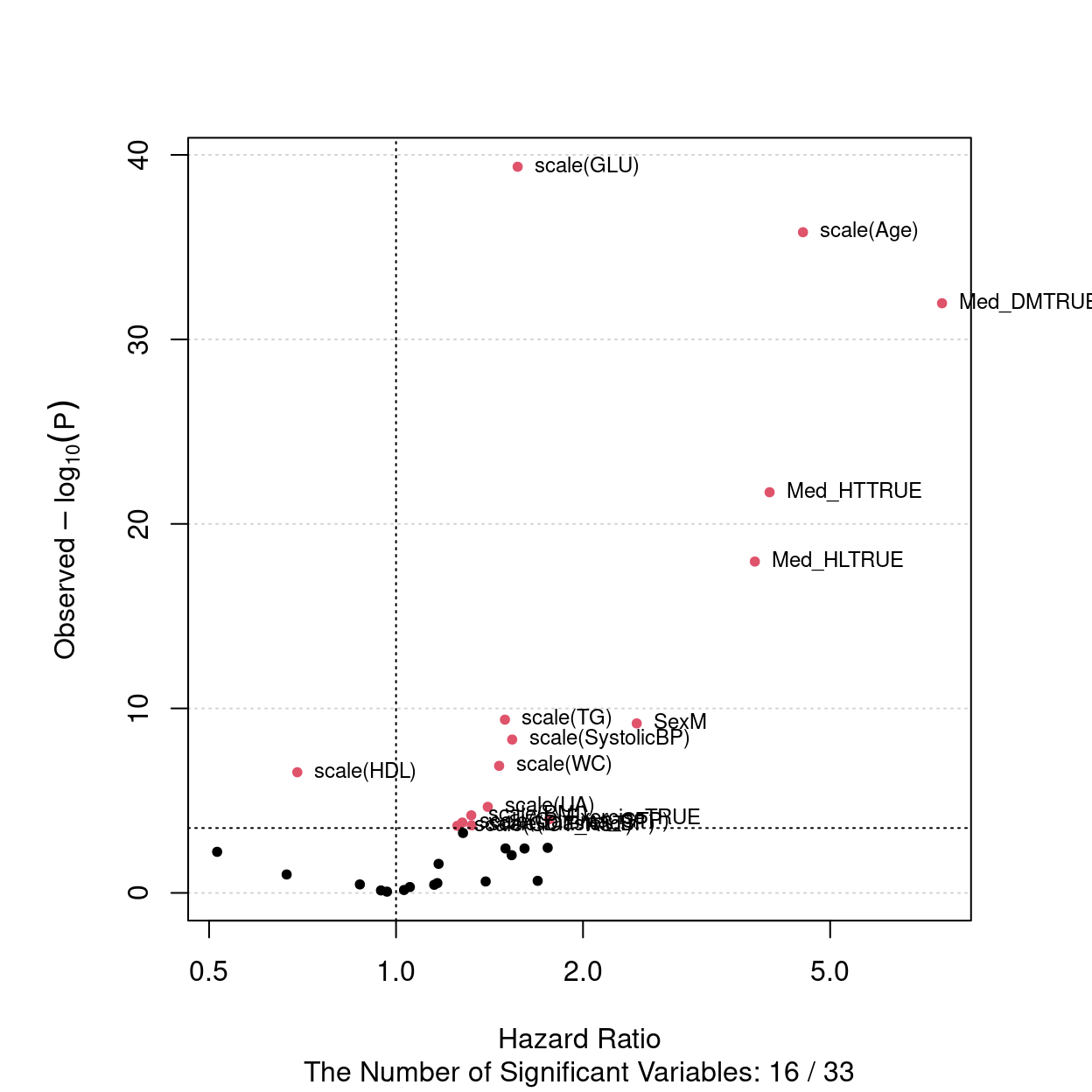
 Appendix Figure 8. Associations between exposure factors and atherosclerosis**

Cox proportional hazards model revealed the exposure factors associated with a particular event at a 1% Bonferroni-corrected level. Hazard ratios and log_10_(P) values were plotted on a volcanic plot. Continuous variables were converted to log space with base 10 before analysis. Continuous variables were scaled such that hazard ratios reflected incremental changes in hazard per 1 standard deviation change in the predictor.

AST, aspartate transaminase; BMI, body mass index; BP, blood pressure; DM, diabetes mellitus; γ-GTP, γ-glutamyl transpeptidase; GLU, fasting plasma glucose; HDL, high-density lipoprotein cholesterol; HT, hypertension; HL, hyperlipidemia; TG, triglyceride; UA, uric acid; WC, waist circumference

**
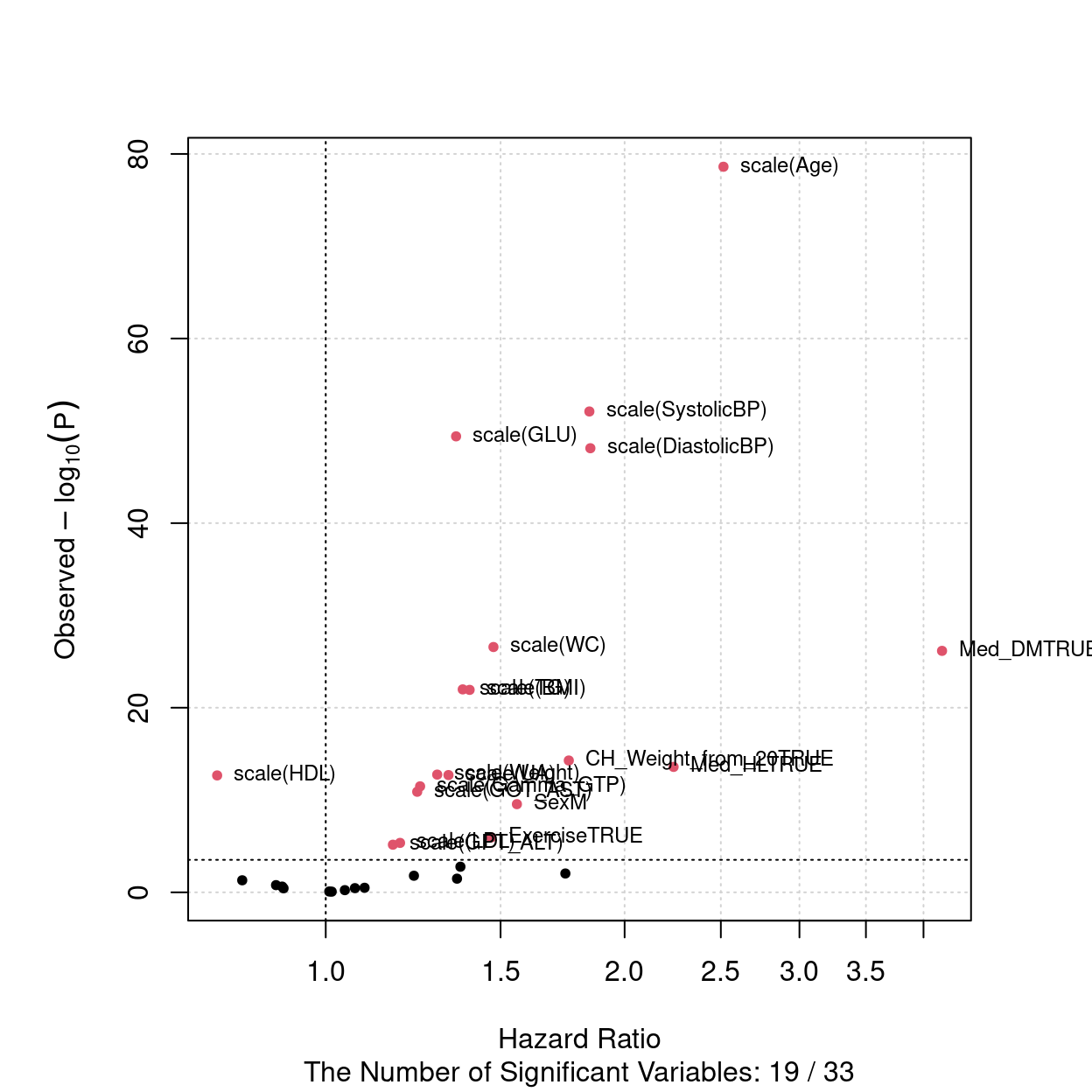
 Appendix Figure 9. Associations between exposure factors and hypertension**

Cox proportional hazards model revealed the exposure factors associated with a particular event at a 1% Bonferroni-corrected level. Hazard ratios and log_10_(P) values were plotted on a volcanic plot. Continuous variables were converted to log space with base 10 before analysis. Continuous variables were scaled such that hazard ratios reflected incremental changes in hazard per 1 standard deviation change in the predictor.

ALT, alanine transaminase; AST, aspartate transaminase; BMI, body mass index; BP, blood pressure; DM, diabetes mellitus; γ-GTP, γ-glutamyl transpeptidase; GLU, fasting plasma glucose; HDL, high-density lipoprotein cholesterol; HT, hypertension; HL, hyperlipidemia; LDL, low-density lipoprotein cholesterol; TG, triglyceride; UA, uric acid; WC, waist circumference

**
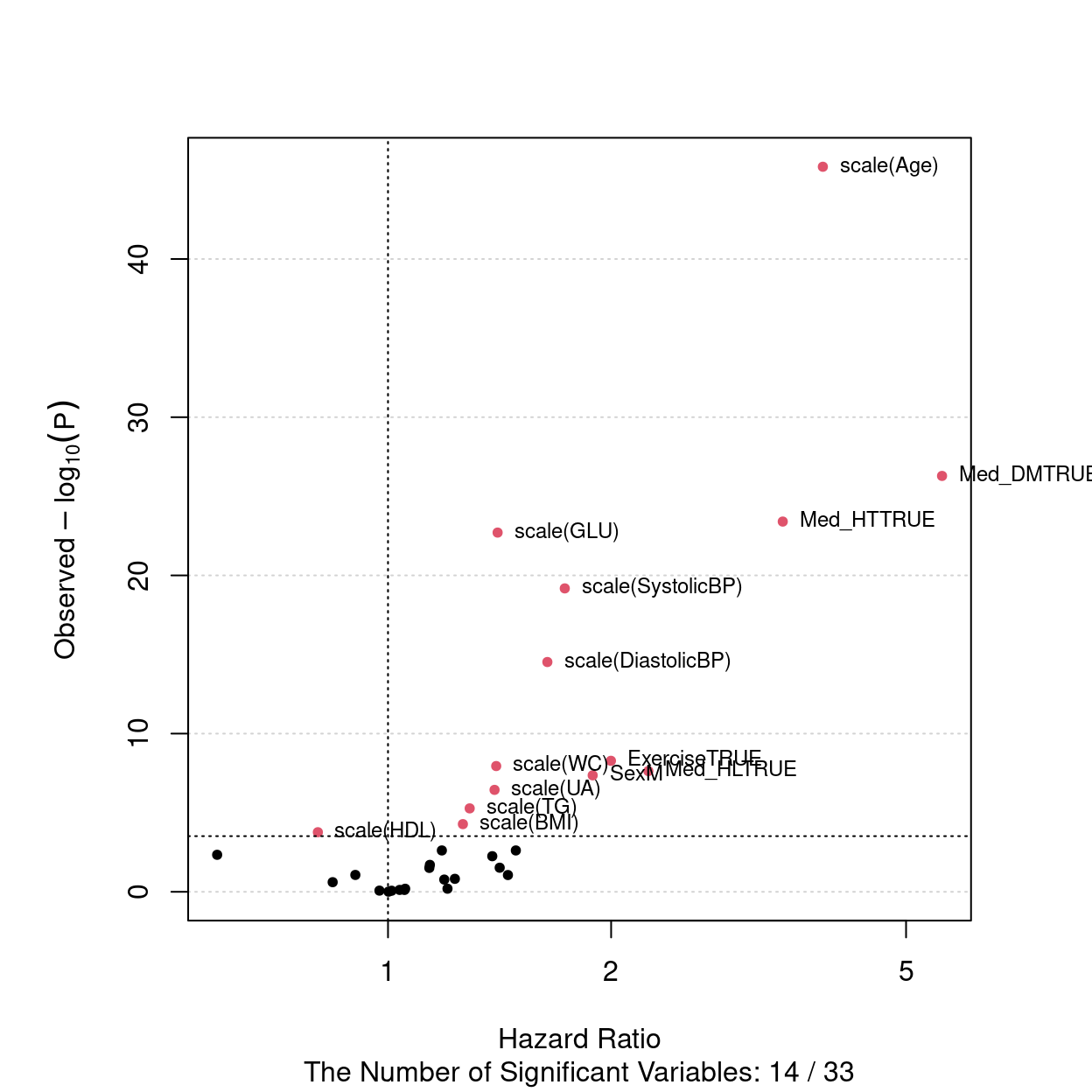
 Appendix Figure 10. Associations between exposure factors and cerebral infarction**

Cox proportional hazards model revealed the exposure factors associated with a particular event at a 1% Bonferroni-corrected level. Hazard ratios and log_10_(P) values were plotted on a volcanic plot. Continuous variables were converted to log space with base 10 before analysis. Continuous variables were scaled such that hazard ratios reflected incremental changes in hazard per 1 standard deviation change in the predictor.

BMI, body mass index; BP, blood pressure; DM, diabetes mellitus; GLU, fasting plasma glucose; HDL, high-density lipoprotein cholesterol; HT, hypertension; HL, hyperlipidemia; TG, triglyceride; UA, uric acid; WC, waist circumference; M, men

**
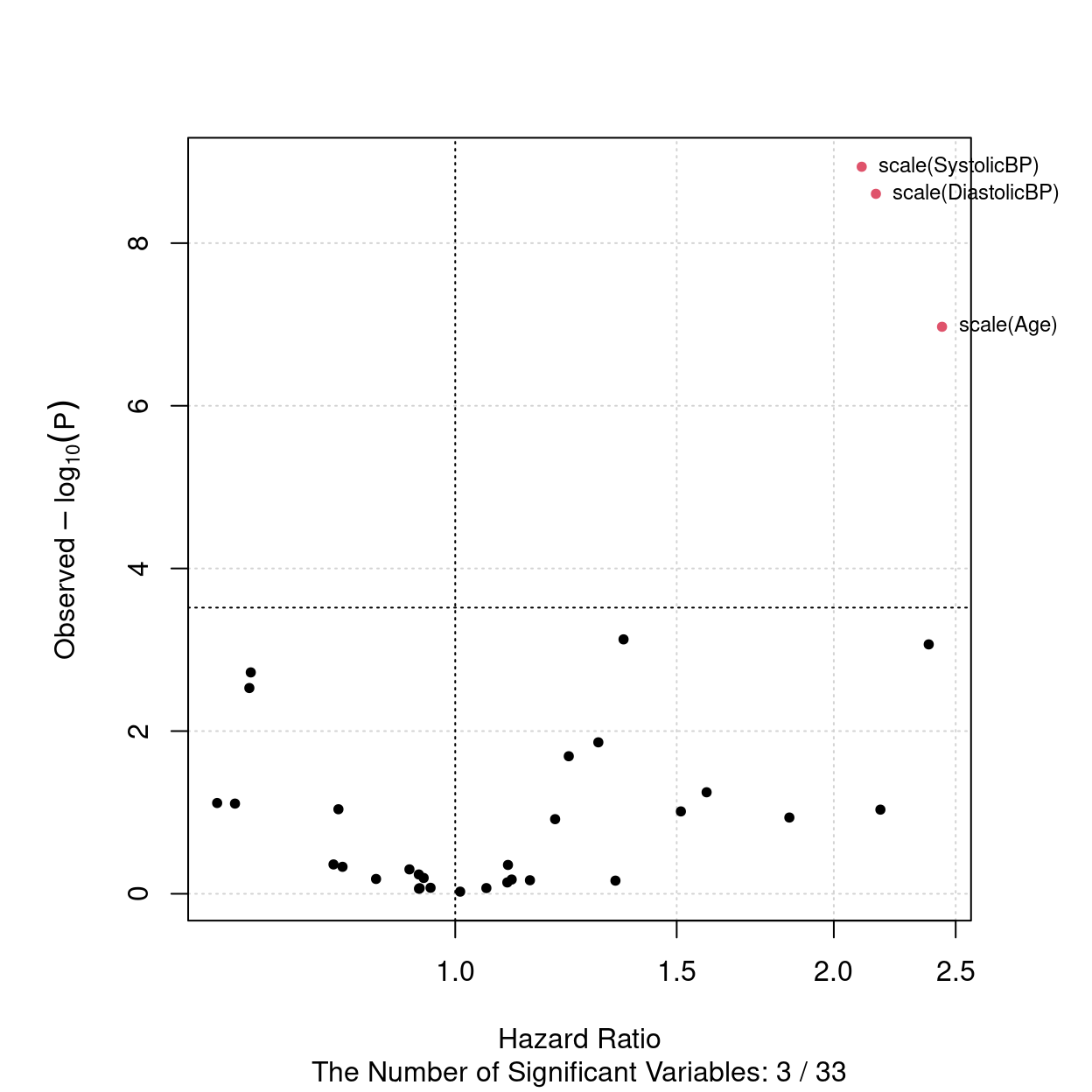
 Appendix Figure 11. Associations between exposure factors and intracerebral hemorrhage**

Cox proportional hazards model revealed the exposure factors associated with a particular event at a 1% Bonferroni-corrected level. Hazard ratios and log_10_(P) values were plotted on a volcanic plot. Continuous variables were converted to log space with base 10 before analysis. Continuous variables were scaled such that hazard ratios reflected incremental changes in hazard per 1 standard deviation change in the predictor.

BP, blood pressure

**
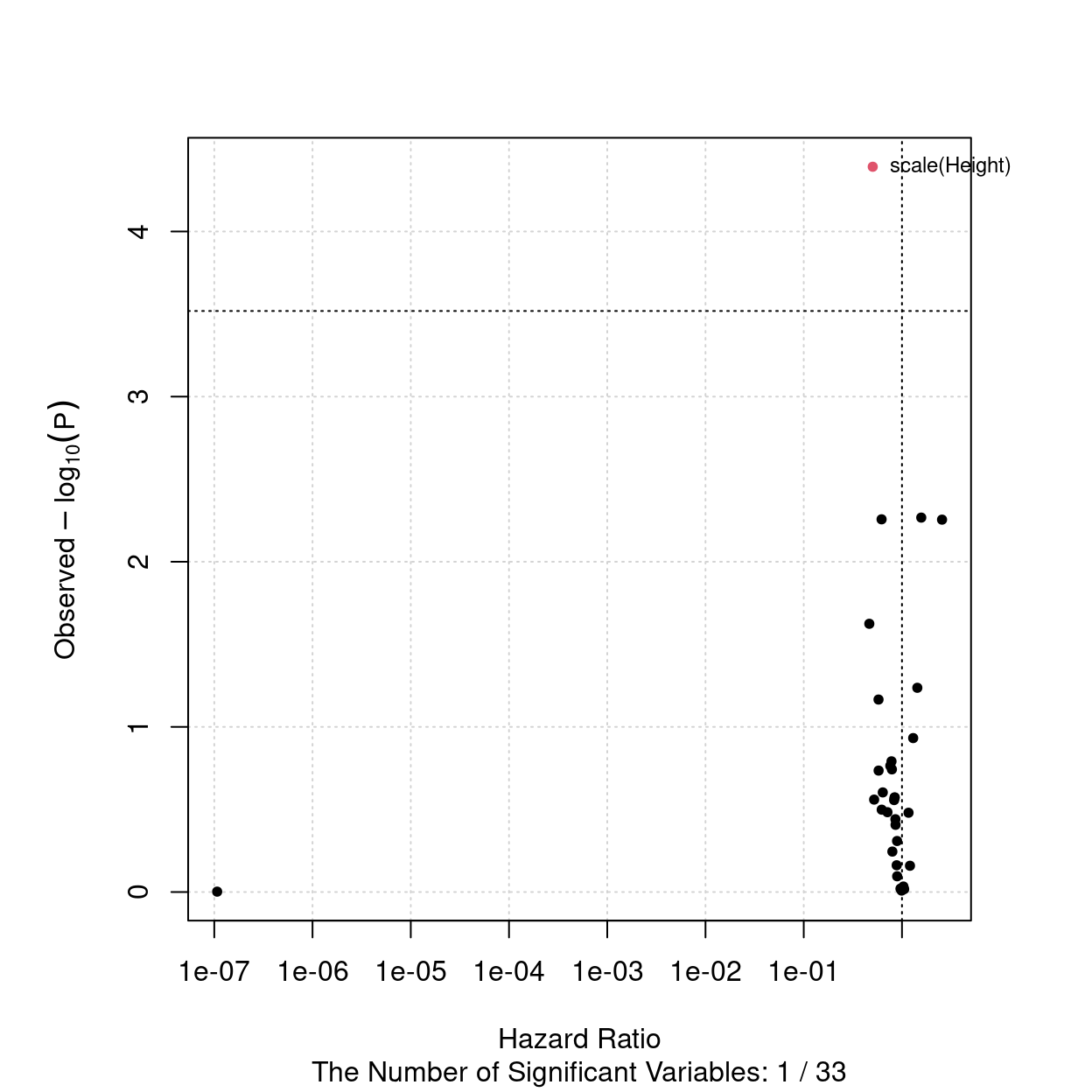
 Appendix Figure 12. Associations between exposure factors and subarachnoid hemorrhage**

Cox proportional hazards model revealed the exposure factors associated with the event at a 1% Bonferroni-corrected level. Hazard ratios and log_10_(P) values were plotted on a volcanic plot. Continuous variables were converted to log space with base 10 before analysis. Continuous variables were scaled such that hazard ratios reflected incremental changes in hazard per 1 standard deviation change in the predictor.

**
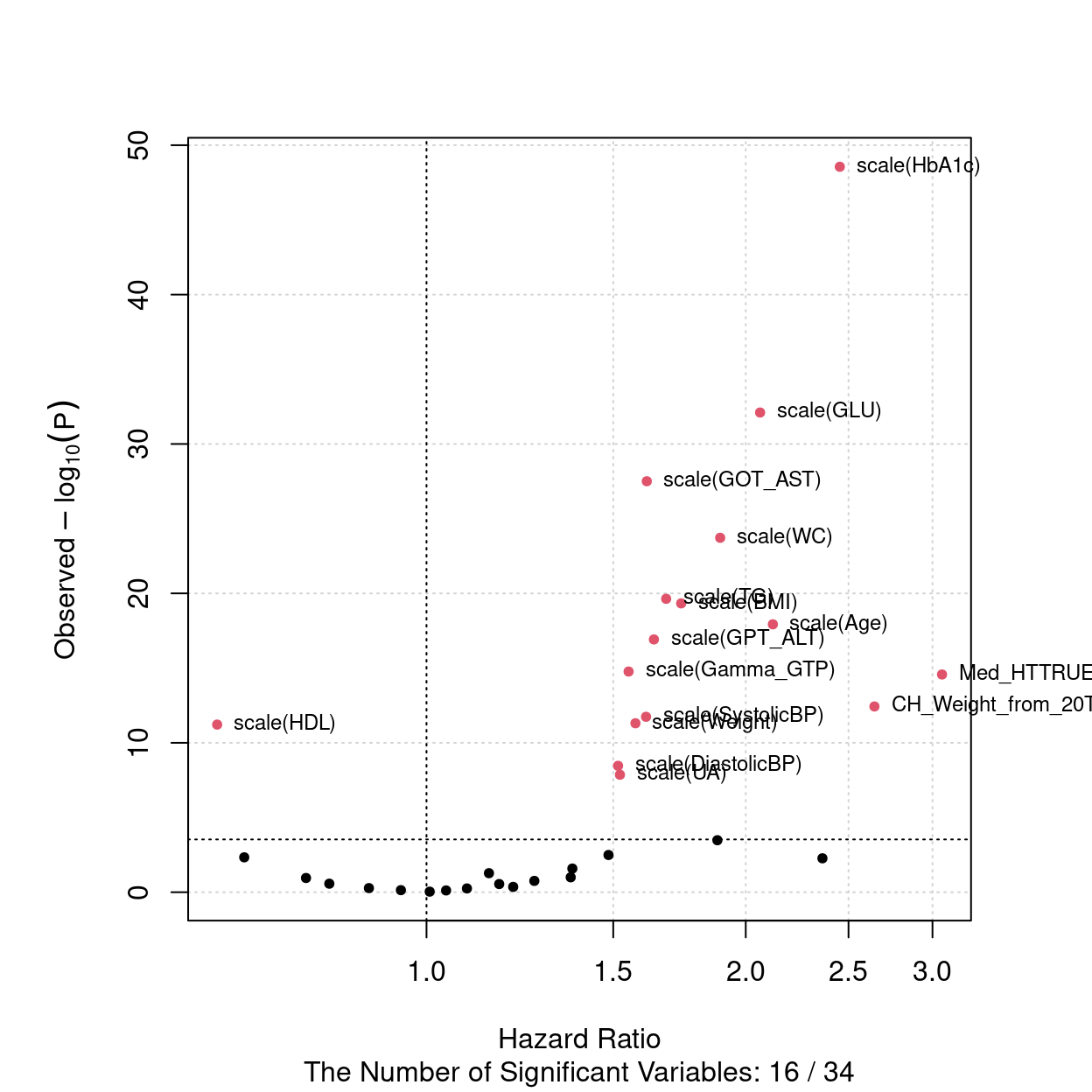
 Appendix Figure 13. Associations between exposure factors and type 2 diabetes mellitus**

Cox proportional hazards model revealed the exposure factors associated with a particular event at a 1% Bonferroni-corrected level. Hazard ratios and log_10_(P) values were plotted on a volcanic plot. Continuous variables were converted to log space with base 10 before analysis. Continuous variables were scaled such that hazard ratios reflected incremental changes in hazard per 1 standard deviation change in the predictor. ALT, alanine transaminase; AST, aspartate transaminase; BMI, body mass index; BP, blood pressure; γ-GTP, γ-glutamyl transpeptidase; GLU, fasting plasma glucose; HbA1c, hemoglobin A1c; HDL, high-density lipoprotein cholesterol; HT, hypertension; TG, triglyceride; UA, uric acid; WC, waist circumference

**
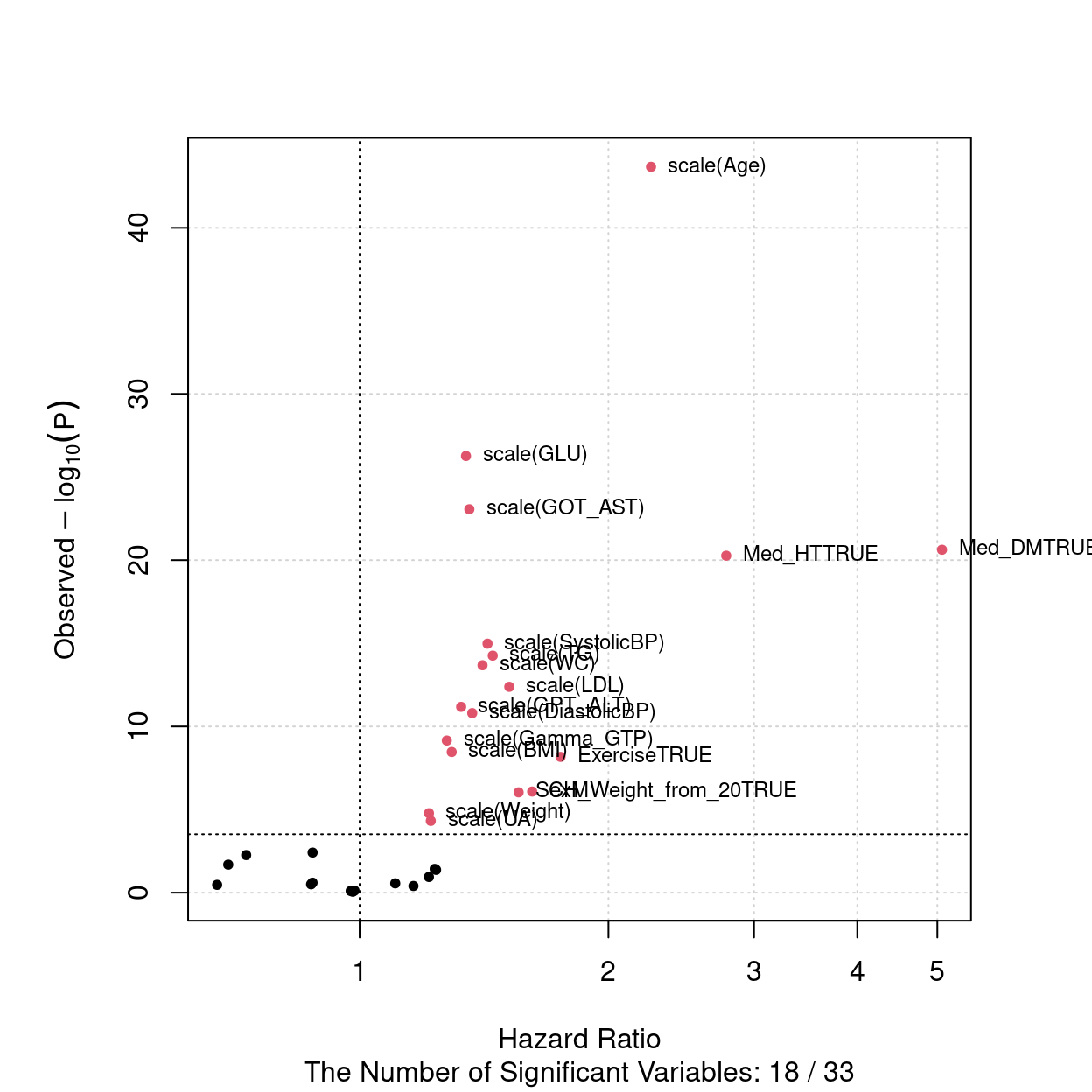
 Appendix Figure 14. Associations between exposure factors and hyperlipidemia**

Cox proportional hazards model revealed the exposure factors associated with a particular event at a 1% Bonferroni-corrected level. Hazard ratios and log_10_(P) values were plotted on a volcanic plot. Continuous variables were converted to log space with base 10 before analysis. Continuous variables were scaled such that hazard ratios reflected incremental changes in hazard per 1 standard deviation change in the predictor. ALT, alanine transaminase; AST, aspartate transaminase; BMI, body mass index; BP, blood pressure; DM, diabetes mellitus; γ-GTP, γ-glutamyl transpeptidase; GLU, fasting plasma glucose; HT, hypertension; LDL, low-density lipoprotein cholesterol; TG, triglyceride; UA, uric acid; WC, waist circumference; M, men

**
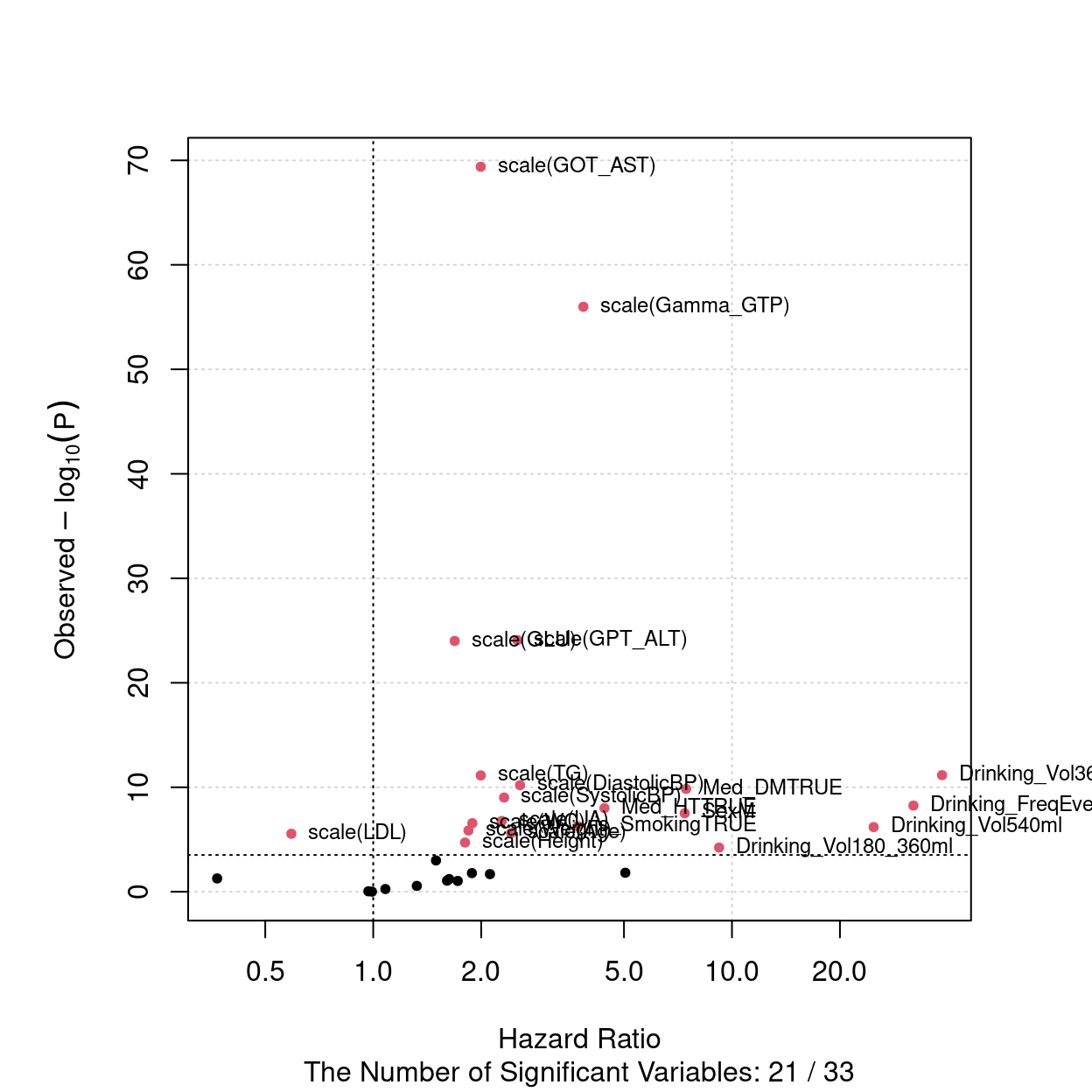
 Appendix Figure 15. Associations between exposure factors and alcoholic liver disease**

Cox proportional hazards model revealed the exposure factors associated with a particular event at a 1% Bonferroni-corrected level. Hazard ratios and log_10_(P) values were plotted on a volcanic plot. Continuous variables were converted to log space with base 10 before analysis. Continuous variables were scaled such that hazard ratios reflected incremental changes in hazard per 1 standard deviation change in the predictor.

ALT, alanine transaminase; AST, aspartate transaminase; BP, blood pressure; DM, diabetes mellitus; γ-GTP, γ-glutamyl transpeptidase; GLU, fasting plasma glucose; HT, hypertension; HL, hyperlipidemia; LDL, low-density lipoprotein cholesterol; TG, triglyceride; UA, uric acid; WC, waist circumference

**
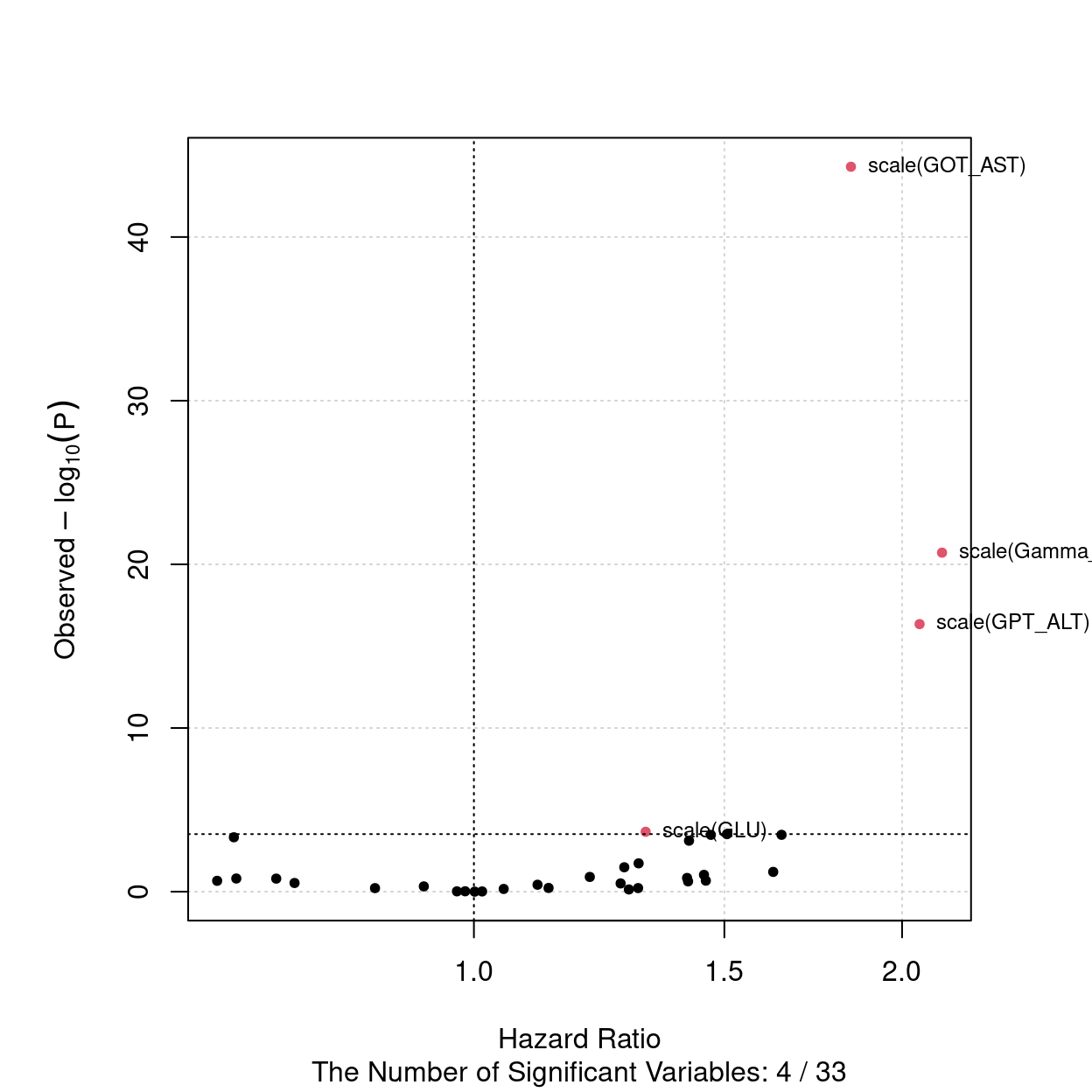
 Appendix Figure 16. Associations between exposure factors and liver fibrosis and cirrhosis**

Cox proportional hazards model revealed the exposure factors associated with a particular event at a 1% Bonferroni-corrected level. Hazard ratios and log_10_(P) values were plotted on a volcanic plot. Continuous variables were converted to log space with base 10 before analysis. Continuous variables were scaled such that hazard ratios reflected incremental changes in hazard per 1 standard deviation change in the predictor.

ALT, alanine transaminase; AST, aspartate transaminase; γ-GTP, γ-glutamyl transpeptidase; GLU, fasting plasma glucose

**
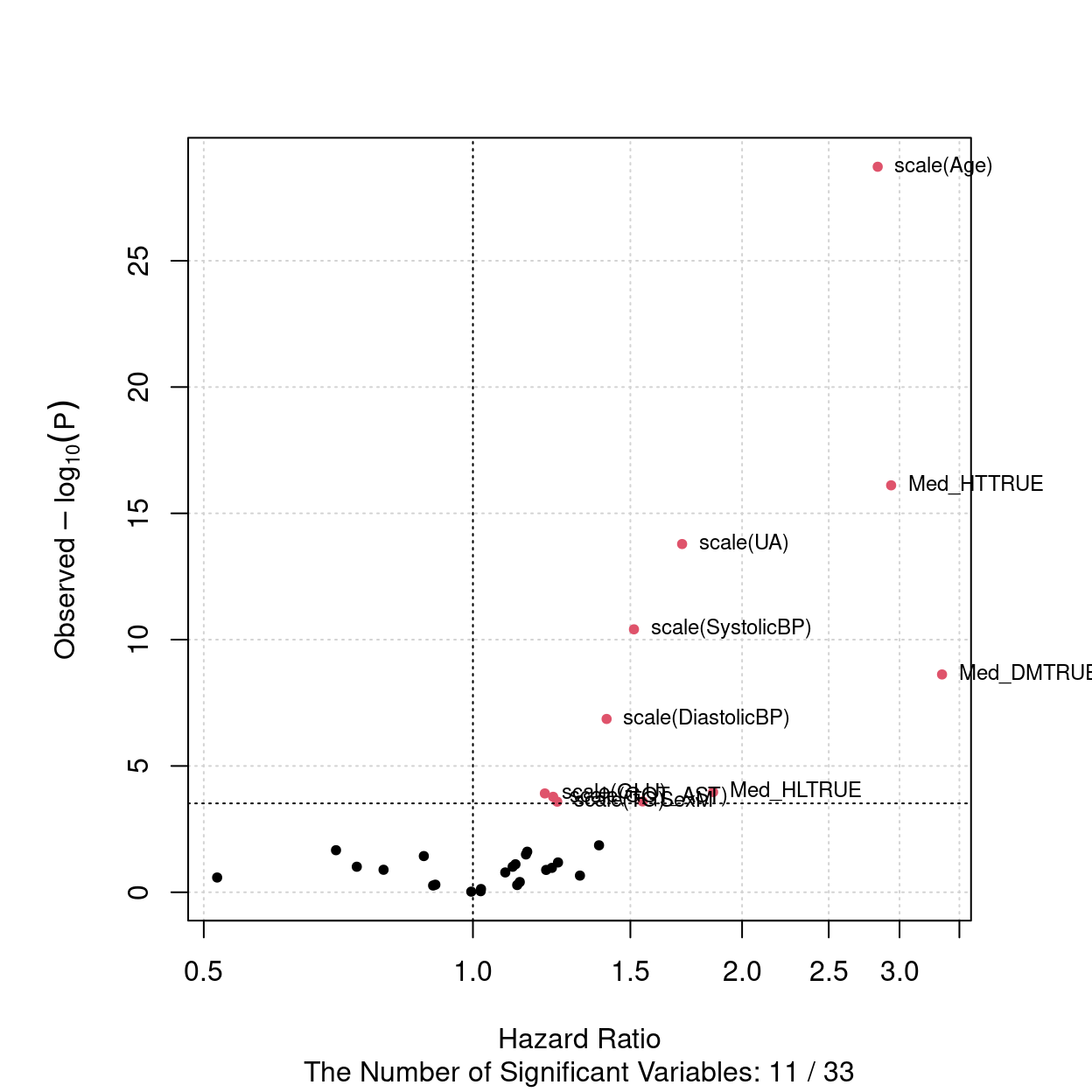
 Appendix Figure 17. Associations between exposure factors and chronic kidney disease**

Cox proportional hazards model revealed the exposure factors associated with a particular event at a 1% Bonferroni-corrected level. Hazard ratios and log_10_(P) values were plotted on a volcanic plot. Continuous variables were converted to log space with base 10 before analysis. Continuous variables were scaled such that hazard ratios reflected incremental changes in hazard per 1 standard deviation change in the predictor.

AST, aspartate transaminase; BP, blood pressure; DM, diabetes mellitus; γ-GTP, γ-glutamyl transpeptidase; GLU, fasting plasma glucose; HT, hypertension; HL, hyperlipidemia; TG, triglyceride; UA, uric acid


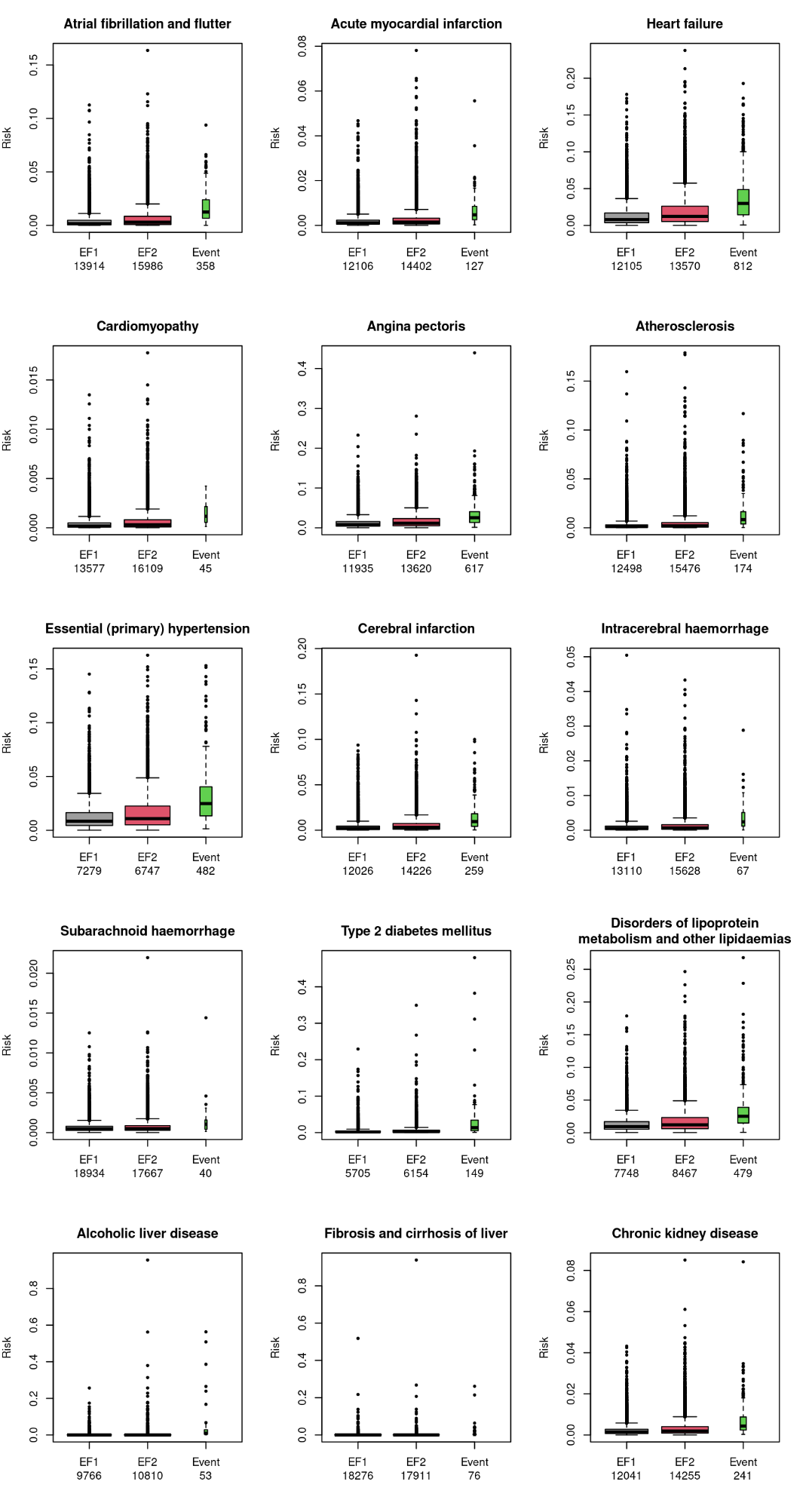


**Appendix Figure 18. The distribution of 4-year risks**

EF1 (Event-free 1): the participants in this group did not have any diagnosis during the period from the first visit for a health checkup to their last visit for a health checkup. EF2 (Event-free 2): the event-free group that follow-up period was defined by their last visit for a health checkup or the latest diagnosis of other diseases, whichever was later.
